# “*Candidatus* Paraporphyromonas polyenzymogenes” encodes multi-modular cellulases linked to the type IX secretion system

**DOI:** 10.1186/s40168-018-0421-8

**Published:** 2018-03-01

**Authors:** A. E. Naas, L. M. Solden, A. D. Norbeck, H. Brewer, L. H. Hagen, I. M. Heggenes, A. C. McHardy, R. I. Mackie, L. Paša-Tolić, M. Ø. Arntzen, V. G. H. Eijsink, N. M. Koropatkin, M. Hess, K. C. Wrighton, P. B. Pope

**Affiliations:** 1 Faculty of Chemistry, Biotechnology and Food Science, Norwegian University of Life Sciences (NMBU), Post Office Box 5003, 1432 Ås, Norway; 2 Department of Microbiology, The Ohio State University, Columbus, OH 43201 USA; 3 Environmental and Molecular Sciences Laboratory, Pacific Northwest National Laboratory, Richland, WA 99354 USA; 4 Computational Biology of Infection Research, Helmholtz Centre for Infection Research, Inhoffenstraβe 7, 38124 Braunschweig, Germany; 5 Institute for Genomic Biology and Department of Animal Sciences, University of Illinois at Urbana-Champaign, Urbana, IL 61801 USA; 6 Department of Microbiology and Immunology, University of Michigan Medical School, Ann Arbor, MI 48109 USA; 7 Department of Animal Science, University of California, Davis, CA 95616 USA

**Keywords:** Type IX secretion system, Carbohydrate-active enzymes, Cellulases, *Bacteroidetes*

## Abstract

**Background:**

In nature, obligate herbivorous ruminants have a close symbiotic relationship with their gastrointestinal microbiome, which proficiently deconstructs plant biomass. Despite decades of research, lignocellulose degradation in the rumen has thus far been attributed to a limited number of culturable microorganisms. Here, we combine meta-omics and enzymology to identify and describe a novel *Bacteroidetes* family (“*Candidatus* MH11”) composed entirely of uncultivated strains that are predominant in ruminants and only distantly related to previously characterized taxa.

**Results:**

The first metabolic reconstruction of Ca. MH11-affiliated genome bins, with a particular focus on the provisionally named “*Candidatus* Paraporphyromonas polyenzymogenes”, illustrated their capacity to degrade various lignocellulosic substrates via comprehensive inventories of singular and multi-modular carbohydrate active enzymes (CAZymes). Closer examination revealed an absence of archetypical polysaccharide utilization loci found in human gut microbiota. Instead, we identified many multi-modular CAZymes putatively secreted via the *Bacteroidetes*-specific type IX secretion system (T9SS). This included cellulases with two or more catalytic domains, which are modular arrangements that are unique to *Bacteroidetes* species studied to date. Core metabolic proteins from Ca. P. polyenzymogenes were detected in metaproteomic data and were enriched in rumen-incubated plant biomass, indicating that active saccharification and fermentation of complex carbohydrates could be assigned to members of this novel family. Biochemical analysis of selected Ca. P. polyenzymogenes CAZymes further iterated the cellulolytic activity of this hitherto uncultured bacterium towards linear polymers, such as amorphous and crystalline cellulose as well as mixed linkage β-glucans.

**Conclusion:**

We propose that Ca. P. polyenzymogene genotypes and other Ca. MH11 members actively degrade plant biomass in the rumen of cows, sheep and most likely other ruminants, utilizing singular and multi-domain catalytic CAZymes secreted through the T9SS. The discovery of a prominent role of multi-modular cellulases in the Gram-negative *Bacteroidetes*, together with similar findings for Gram-positive cellulosomal bacteria (*Ruminococcus flavefaciens*) and anaerobic fungi (*Orpinomyces* sp.), suggests that complex enzymes are essential and have evolved within all major cellulolytic dominions inherent to the rumen.

**Electronic supplementary material:**

The online version of this article (10.1186/s40168-018-0421-8) contains supplementary material, which is available to authorized users.

## Background

Ruminants are herbivorous mammals that constitute approximately 200 different species, including 3.5 billion domesticated animals that produce important commodities [1]. The rumen is an archetypal plant biomass-degrading ecosystem, which harbors an elaborate microbiome that has been extensively studied to obtain a mechanistic understanding of microbial lignocellulose degradation. Despite significant efforts over many decades, rumen cellulose degradation has so far been attributed to a limited number of culturable representatives, including *Firmicutes* and *Fibrobacteres* species. The Firmicute *Ruminococcus flavefaciens* utilizes endo- and exocellulase-containing cellulosomes for the degradation of highly recalcitrant substrates [2]. *Fibrobacter succinogenes* in turn uses an enigmatic mechanism that does not involve cellulosomes or known exocellulases implicated in secreted cellulase systems [3]. Instead, *F. succinogenes* is believed to employ fibro-slime and pili proteins to adhere to cellulose and convert the substrate to cellodextrins by secreted endo-cellulases that are anchored to the outer membrane of cells [4] as well as outer membrane vesicles [5]. Whereas the contribution of the *Bacteroidetes* to hemicellulose and pectin degradation within the rumen is well established, evidence for cellulose degradation by this phylum remains limited [6, 7]. An aerobic soil Bacteroidete, *Cytophaga hutchinsonii*, has been suggested to use a non-classical cellulolytic mechanism during which the organism adheres closely to cellulose and glides across the substrate, degrading it with periplasmic and extracellular endocellulases that are secreted by the type IX secretion system (T9SS) [8].

Metagenomic techniques have increased knowledge of the uncultured lineages that dominate digestive ecosystems. In 2011, an ultra-deep metagenomic sequencing project from a switchgrass adherent community generated 268 Gbp of shotgun sequence data and recovered 446 population genome bins, 15 of which were greater than 60% complete [9]. These 15 draft genomes were systematically examined in more detail in our laboratories using several bioinformatic approaches [10, 11], which suggested that five of the 15 genomes are representative of uncultivated cellulose-degrading lineages. This refined list included the genome bin denoted as AGa, which is the focus of this study. Here, we report a detailed characterization of AGa, an uncultured *Bacteroidetes*-affiliated genotype that is predicted to be cellulolytic, albeit via an unknown mechanism. Based on sequencing (305 ± 33 coverage), AGa was the second most abundant assembled genome, and its 3.08 Mb genome was estimated to be ~ 90% complete, with less than 4% contamination [9]. Our data reveals that AGa constitutes one of the seven genomes within a novel family referred to as “*Candidatus* MH11”. Importantly, the AGa genome and several other Ca. MH11 representatives encode multi-modular cellulases that have not been previously described for *Bacteroidetes*-affiliated species [12]. We also present metaproteomic evidence of AGa proteins in fiber-adherent microbiomes as well as biochemical profiles of AGa carbohydrate-active enzymes (CAZymes) acting on cellulose. Taking meta-omics and enzymology analyses into consideration, we propose a non-classical cellulose degradation mechanism that involves the utilization of multi-modular enzymes that are secreted via the *Bacteroidetes*-specific T9SS.

## Results

### Phylogenomics and genome reconstruction resolve the novel family Ca. MH11 and identify “*Candidatus* Paraporphyromonas polyenzymogenes”

**Fig. 1:**
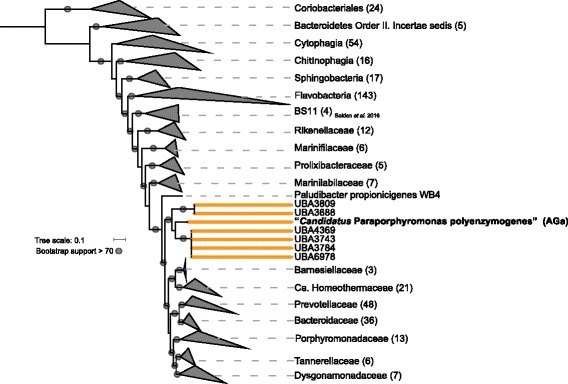
Concatenated ribosomal protein tree of the AGa genome and genomic representatives from the phylum *Bacteroidetes*. The *Coriobacteriales* within the *Actinobacteria* are also included as an outgroup. Clades are collapsed by family with the number of genomes denoted in parentheses next to the name. Family names follow the description by Ormerod et al. [62]. The newly proposed Ca. MH11 is highlighted in orange. Grey circles on the node represent bootstrap support > 70, using 100 bootstraps

In order to resolve the taxonomy of AGa, we performed phylogenetic analyses using concatenated alignments of 16 single-copy ribosomal proteins (Fig. 1). To assist in the phylogenetic context, we also recruited near neighbors from the recently created Uncultured Bacteria and Archaea (UBA) database, which contains 7903 assembled high-quality draft genomes, including many from ruminant metagenomes [13]. Our analysis indicated that AGa falls within a monophyletic family composed of six sheep rumen associated UBA genomes within the order *Bacteroidales* in the *Bacteroidetes* phylum (Fig. 1). Further support for placing AGa into a newly resolved family, here denoted as Ca. MH11, was provided by other single marker gene analyses and a 16S rRNA gene fragment within one of seven genome bins (UBA3809) that was closely related to AGa (Fig. 1). Sequence-based genome comparisons illustrated that AGa has < 70% average nucleotide identity (ANI) and < 55% average amino acid identify (AAI) to all reference genomes selected across the known families in the *Bacteroidales* (Additional file 1: Figure S1). This further validated the designation of the new Ca. MH11 family.

**Fig. 2:**
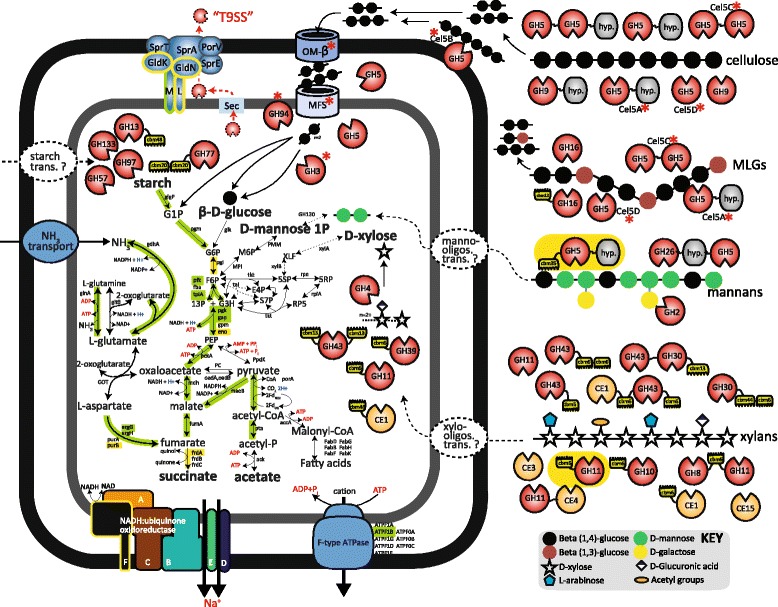
Selected metabolic features of “*Candidatus* Paraporphyromonas polyenzymogenes” (AGa) as inferred from genome and proteome comparisons. Graphical representation of pathways, enzymes, CAZymes, and cellular features are based on functional annotations listed in Additional file 2: Table S1. Ca. P. polyenzymogenes encoded glucose, mannose, and xylose fermentation capabilities, as well as CAZymes inferred in degradation of cellulose, mannans, and xylans. CAZymes located outside the cell are predicted to be secreted via the T9SS. Metaproteomic analysis (detected genes, enzyme complexes, and transport systems highlighted in green) indicated that Ca. P. polyenzymogenes was metabolically active in rumen samples, with proteins for glycolysis, succinate production, and ammonium metabolism detected, as well as components of the T9SS and T9SS-secreted multimodular CAZymes (details found in Additional file 2: Table S1, Additional file 7: Table S4). Features highlighted by yellow boxes indicate detected genes that were identified in only one sample (Additional file 7: Table S4). Hypothetical domains in multi-modular CAZymes are denoted by “hyp.”, and the red asterisks indicate CAZymes that were encoded in the gene cluster characterized in this study (Fig. 3). Broken lines indicate annotations for which representative genes were not identified in the respective reconstructed genomes. Further details on enzyme abbreviations, gene abbreviations, and identification numbers (Integrated Microbial Genomics gene ID) can be found in Additional file 2: Table S1. The abbreviations for central metabolites are PEP phosphoenolpyruvate, G1P glucose-1-phosphate, G6P glucose-6-phosphate, F6P fructose-6-phosphate, M6P mannose-6-phosphate, G3H d-glyceraldehyde 3-phosphate, E4P d-erythrose 4-phosphate, 5SP d-xylulose 5-phosphate, S7P sedoheptulose 7-phosphate, 5RP d-ribulose 5-phosphate, and RP5 d-ribose 5-phosphate

AGa was numerically abundant within a switchgrass-associated microbiome that was incubated within the cow rumen for 72 h and which showed substantial carbohydrate degradative capabilities [9]. Switchgrass is a promising lignocellulosic bioenergy crop that is predominated by cellulose and xylan hemicellulose and thus contains high amounts of glucose and xylose monomers (average 38 and 22.8%, respectively) [14]. As expected, a comprehensive metabolic reconstruction of AGa predicted extensive polysaccharide-degrading capabilities, with more than 100 AGa genes containing at least one CAZyme domain. AGa genes inferred in the hydrolysis of cellulosic substrates included 17 putative endocellulases (glycosyl hydrolase (GH) family: GH5 and GH9) as well as GH3 β-glucosidases and GH94 cellobiose phosphorylases that convert shorter cello-oligosaccharides into monomeric glucose (Fig. 2, Additional file 2: Table S1). Consistent with other *Bacteroidetes*-affiliated genomes, no known exocellulases (GH6, GH48) or cellulosomal components (i.e. cohesins, dockerins) were identified. Additionally, many of the putative CAZymes identified in AGa were predicted to be involved in the deconstruction of non-cellulosic polysaccharides prevalent in the cell walls of grasses. Predicted enzymes suggest AGa to be capable of hydrolyzing various xylan and arabinoxylan moieties, which are a major constituents of switchgrass [14, 15]. The AGa genome encoded presumptive endoxylanases (GH10 and GH11), β-xylosidases (GH3, GH30, GH39 and GH43), α-L-arabinofuranosidases (GH51), α-glucuronidases (GH4) and acetyl xylan carbohydrate-esterases (CE1, CE3 and CE4). Genes encoding CAZyme domains inferred in the conversion of mixed-linkage glucans (GH16), xyloglucans (GH74), mannans (GH26) and starch (GH13, GH57, GH97, GH133) were also identified. This CAZyme profile was relatively consistent within all Ca. MH11 representatives (Additional file 3: Table S2), suggesting that similar saccharolytic capabilities are shared within this unique *Bacteroidales* clade.

Despite evidence that AGa is a saccharolytic member of the *Bacteroidales*, our analysis revealed that the genome is devoid of archetypical components constituting polysaccharide utilization loci (PUL), i.e. gene clusters encoding both SusC/SusD-like lipoproteins and CAZymes [16, 17]. This was unexpected, as the polysaccharide-degrading capacity of gut-associated *Bacteroidetes* described to date has been attributed to their PUL-based systems [18]. As an alternative of PUL-associated CAZymes, a total of 42 AGa genes were found to encode a CAZyme domain and an additional conserved carboxy-terminal domain (CTD) that functions as an outer membrane translocation signal for export via the T9SS (Additional file 2: Table S1) [19]. Correspondingly, the necessary T9SS components were also encoded within the AGa genome [20, 21], indicating that AGa exports T9SS-CTD containing CAZymes that target cellulose and hemicellulose (Fig. 2, Additional file 2: Table S1). Scenarios similar to this hypothesis also occur in the aerobic cellulolytic soil Bacteroidete *Cytophaga hutchinsonii*, where it has been shown that non-PUL mechanisms are employed [22], instead using the T9SS for exporting five different endoglucanases that contribute to cellulose utilization [8, 23]. However, as the rumen is anaerobic, representatives of *C. hutchinsonii* are predictably absent.

Many of the T9SS-secreted CAZymes encoded in the AGa genome exhibit a multi-modular arrangement, and as commonly observed, many of these CAZymes contain one or more carbohydrate-binding modules (CBMs). More interestingly, several of the identified CAZymes contain multiple catalytic domains, including various cellulases (GH5-GH5), xylanases (GH8-CBM6-GH11) and mannanases (GH5-GH26) (Additional file 4: Figure S2, Additional file 2: Table S1). In addition, several genes encoded one or more GH domains as well as a flanking hypothetical region for which no known GH or CBM annotation has been assigned (Additional file 4: Figure S2). Examination of the other Ca. MH11 representatives from the sheep rumen revealed similar T9SS-secreted multi-modular CAZymes including unique putative ORFs that are ~ 1900 amino acids in length and encode six consecutive GH5 domains (Additional file 4: Figure S2). Examples of operon-like gene clusters that encompasses T9SS-secreted CAZymes were also observed in AGa and other Ca. MH11 representatives, which are predicted to target both cellulosic (Additional file 5: Figure S3) and hemicellulosic substrates (Additional file 6: Table S3). Genes encoding multiple catalytic CAZyme domains are less frequently observed in microbial genomes; however, recent studies have illustrated their profound impact in polysaccharide hydrolysis. The exemplar cellulase *Cb*CelA from *Caldicellulosiruptor bescii* (GH9-CBM3c-CBM3b-CBM3b-GH48) has been demonstrated to outperform mixtures of commercial exo- and endocellulases, likely due to its inter-domain synergy [24]. Similarly, a highly efficient chitinase from the Bacteroidete *Flavobacterium johnsoniae* (*Fj*ChiA) comprises an exo- and an endo-acting GH18 domain and domains with substrate affinity [25]. *Fj*ChiA also contains a CTD and has been shown to be secreted via the T9SS, whereas gene knock-out mutagenesis has demonstrated that the enzyme is vital for chitin metabolism and cell growth [26]. Non-covalent multi-modular cellulase arrangements are known to contribute to biomass conversion in the rumen, as exemplified by the ruminant Firmicute *R. flavefaciens*, which uses a complex cellulosome for polysaccharide hydrolysis [12, 27]. Interestingly, several genes encoding multiple GH5 domains have also been identified in the cellulose-degrading rumen fungi *Orpinomyces* sp. [28], whereas multi-modular CAZymes are not common in aerobic fungi. Synthesizing these findings, it seems reasonable to hypothesize that a saccharolytic strategy based on employing the power of multi-modular CAZymes has evolved in ruminant *Bacteroidetes*, such as AGa, in coordination with the phylum-specific T9SS that allows for export of large proteins [29].

Although AGa is predicted to possess broad hydrolytic capabilities outside the cell, known transporters for starch-, mannan- and xylan-derived oligosaccharides were surprisingly not detected (Fig. 2). Possible explanations include (I) the presence of unknown transport systems, (II) the relevant genes being contained in the missing fraction of the AGa genome (< 10%), or (III) AGa is degrading complex carbohydrates extracellularly but not consuming the immediate hydrolysis products. Monomeric hexoses (glucose and mannose) were predicted to be catabolized via the Embden-Meyerhof-Parnas (EMP) pathway (Fig. 2). Whilst AGa contained CAZymes to deconstruct xylose-containing polysaccharides, only a partial pentose phosphate pathway was present, and there was no evidence for the presence of the xylose isomerase pathway. Closer inspection of the EMP pathway could not identify a putative pyruvate kinase that catalyzes phosphoenolpyruvate (PEP) to pyruvate. Instead, we predict that AGa employs a branched, rather than complete, tricarboxylic acid cycle that uses an anaplerotic reaction to produce oxaloacetate (OAA) from PEP, with the subsequent reduction of OAA to succinate [30]. Additionally, genes encoding putative oxaloacetate decarboxylase subunits were found (OAA → pyruvate). The production of acetate to generate ATP via substrate-level phosphorylation was also predicted by the identification of a putative acetate kinase (Fig. 2). The production of both acetate and succinate as principal fermentation end products indicate that AGa is an important rumen symbiont, as both fatty acids are key nutritional requirements of ruminants [31]. Similar to rumen *Prevotella* species, AGa is predicted to assimilate ammonium via genes encoding putative enzymes involved in ammonium uptake, assimilation and its regulation [32].

Metaproteomic analysis was performed on rumen samples that were collected from animals using an experimental design that was consistent with the original AGa-containing metagenome. Briefly, metaproteome datasets were obtained from microbial communities associated with *in situ* incubated switchgrass and corn stover and from the microbiome associated with the bulk rumen fluid of two fistulated cows. Both secreted and cellular AGa proteins of various metabolic functions were detected in all examined samples, with slightly higher detection levels in the proteomes associated with rumen-incubated biomass (Additional file 7: Table S4), reiterating AGa’s importance to rumen microbial communities. Closer examination of the detected AGa proteins in these rumen samples supported functional genomic interpretations; genes in key metabolic pathways identified by metagenomics were expressed in the rumen (Additional file 7: Table S4, Fig. 2). In particular, proteins for glycolysis, succinate production and ammonium metabolism were detected, as well as components of the T9SS and T9SS-secreted multimodular CAZymes inferred in hemicellulose (GH11-CBM6, hypothetical domain-GH5-CBM35) hydrolysis (Fig. 2). As expected, T9SS-secreted CAZymes were detected in biomass-associated samples (Additional file 7: Table S4). Several hypothetical proteins were also widely detected in both rumen fluid and plant-associated samples (Additional file 7: Table S4), reiterating that important undiscovered metabolic functions still remain to be elucidated in rumen microbes.

With both phylogeny and predicted functions considered, we propose the candidate genus name “*Candidatus* Paraporphyromonas” for the AGa clade; the proposed name combines Para (“beside” or “next to”, Greek) and *Porphyromonas*, the original genus from which the T9SS was discovered [33]. We recommend the provisional name “*Candidatus* Paraporphyromonas polyenzymogenes” for the AGa genotype, which combines poly (“many” or “much”, Greek), enzym (“leavened”, Greek) and -genes (“to produce”, Greek).

### Ca. P. polyenzymogenes encodes a cellulolytic gene cluster with four GH5 endoglucanases exhibiting various modular architectures

**Fig. 3:**
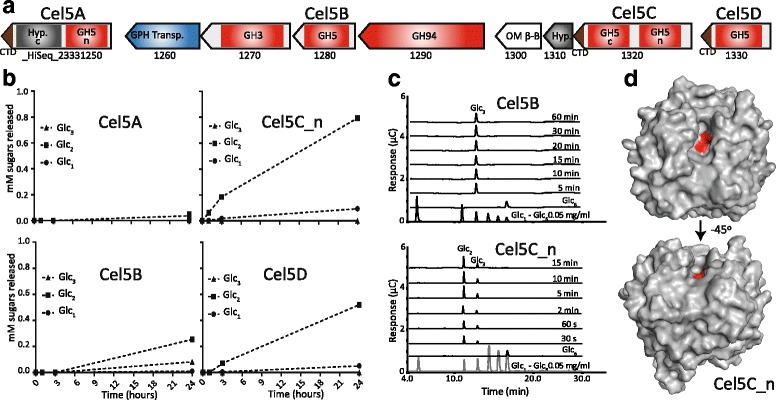
Ca. P. polyenzymogenes encodes a cellulolytic gene cluster. **a** Gene organisation of a putative cellulolytic gene cluster encoding four GH5s with varying modular arrangements. For multi-modular ORFs, both C-terminal (c) and N-terminal (n) domains are indicated, whereas “CTD” denotes a carboxy-terminal domain that infers export via T9SS. Hyp indicates domains with no known function. **b** Products released from crystalline cellulose (Avicel) by Cel5A, Cel5B, Cel5C_N, and Cel5D enzymes. A total of 1% (*w*/*v*) Avicel was incubated with 1 μM enzyme in 20 mM citrate buffer, pH 5.5 at 40 °C with 1000 rpm horizontal shaking. Products were analyzed by HPAEC-PAD at given time points after stopping the reactions by addition of NaOH to 0.1 M. Error bars represent standard deviations between three replicates (hidden by the markers). **c** Degradation of Glc(6) by Cel5B and Cel5C_N enzymes, illustrating differences in substrate specificity. Glc(6) (0.1 mg/ml) was incubated with 0.25 μM enzyme in 20 mM citrate buffer pH 5.5. Samples were taken at indicated intervals, and the reaction was stopped by adding NaOH to 0.1 M. Products were analyzed using HPAEC-PAD with cellodextrins as standards. A more complete analysis for all four cellulases shown in Additional file 13: Figure S7. **d** Crystal structure of Cel5C_N. Surface view of the structure, seen from above the active-site cleft, and − 45° rotated. The surface of the catalytic residues is shown in red. Figures were created using The PyMOL Molecular Graphics System, Version 1.3 Schrödinger, LLC

Gene organization of Ca. P. polyenzymogenes CAZymes revealed an intriguing gene cluster with four putative GH5 cellulases (Cel5A-D) of varying domain architecture, three of which (Cel5A, Cel5C and Cel5D) contain T9SS-CTDs, inferring export outside the cell (Fig. 3a). The single domain Cel5B was predicted to be translocated through the SpII pathway and attached to the outer membrane as a lipoprotein. Cel5A contains a flanking hypothetical region including a truncated CBM6 with low coverage and sequence similarity to characterized CBM6s (< 40% id), which could potentially be a member of a novel CBM family. Cel5C contains two GH5 domains (Cel5C_N and Cel5C_C) that have 45% sequence identity to each other, and low similarity to their closest homologues, which consisted of individual GH5 domains (from single GH-containing proteins) from the known cellulolytic *Bacteroidetes* species *Sporocytophaga myxococcoides* (40–50% sequence identity) and *Saccharicrinis fermentans* (38–56% sequence identity). Cel5D contains a single GH5 domain that exhibits 72% sequence identity to Cel5C_N.

All five GH5 domains in this cluster were affiliated to GH5 subfamily 2, the largest of the GH5 subfamilies [34]. Enzymes in this family are mostly extracellular and are endo*-*acting β-1,4-glucanases. The gene cluster also encodes a GH94 cellobiose phosphorylase and a GH3 β-glucosidase, which were predicted to be cytoplasmic and periplasmic membrane bound, respectively. Two putative transport genes were also identified including a glycoside-pentoside-hexuronide (GPH) cation symporter, which is inferred to uptake sugars in symport with monovalent cations in the inner bacterial membrane. Another possible transporter protein (HiSeq_23331300) was predicted to be an outer membrane β-barrel protein [35]. Finally, a putative lipoprotein with unknown function is encoded by a gene located downstream from Cel5C. Collectively, the enzymatic machinery encoded by the cluster could degrade cellulose and import its products, where the GH5 enzymes would release cellodextrins for further degradation to glucose and glucose-1-phosphate by the GH3 and GH94 enzymes.

### GH5 enzymes from the Ca. P. polyenzymogenes cellulase gene cluster are strict β-1,4-glucanases acting on cellulose and linear hemicellulose polysaccharides

To further characterize Ca. P. polyenzymogenes, we biochemically interrogated the GH5 modular arrangements encoded in the aforementioned gene cluster. Various genes encoding only the catalytic domain or full length proteins were synthesized for Cel5A-D and expressed in *E. coli*, followed by protein purification. As expected, the Cel5A-D enzymes and their individual domains demonstrated endocellulase activity on the soluble cellulose analogue carboxymethyl-cellulose (CMC) (Additional file 8: Table S5). Comparing specific activities revealed Cel5D and the individual N-terminal GH5 domain in Cel5C (Cel5C_N) as the most active (Additional file 8: Table S5). Interestingly, Cel5C_N outperformed the full-length Cel5C_wt, whereas the other GH5 domain, Cel5C_C, had too low activity on CMC to determine the specific activity. Cel5C_C was suspected to contain a mutation in its active site (Additional file 9: Figure S4); however, “corrected” versions of this domain (Cel5C_CR) and the full-length enzyme (Cel5C_R) still exhibited low activity (Additional file 10: Supplemental Text S1, Additional file 11: Figure S5)*.* Although Cel5C_C and Cel5C_CR both have catalytic activity, many questions remain when it comes to the functionality of this domain (Additional file 10: Supplemental Text S1) that could not be resolved within the framework of this study. Thus, all further work on the very active Cel5C was done with Cel5C_N. The lipo-anchored Cel5B enzyme also demonstrated relatively low CMCase activity, approximately 20-fold lower than Cel5C_N. Cel5A_wt and its N-terminal GH5 domain (Cel5A_N) displayed low activities on CMC, with approximately the same specific activity for the catalytic domain and full-length enzyme, suggesting that only the GH5 domain is catalytically active.

The C-terminal domain of Cel5A (Cel5A_C), which could be a novel CBM, was catalytically inactive on both CMC and hemicelluloses. Binding experiments showed binding to Avicel, supporting the notion that this hypothetical domain is a cellulose-binding CBM that could promote binding of Cel5A to insoluble cellulose (Additional file 12: Figure S6). Interestingly, sequence comparisons revealed that domains homologous to the Cel5A_C domain occur in other putative Ca*.* P. polyenzymogenes cellulases that contain both GH5 (HiSeq_05874540, HiSeq_23331410) and GH9 domains (HiSeq_15059240) (Additional file 4: Figure S2), suggesting this putative CBM is broadly distributed.

Cel5A-D were also active on crystalline cellulose (Avicel) over a 24-h period (Fig. 3b). Similar to the relative activities on CMC, Cel5C_N and Cel5D were the most active, releasing mostly cellobiose and some glucose from Avicel. The activities of Cel5C_N and Cel5D are comparable to, or higher than the activity of characterized endoglucanases from the cellulose-degrading soil Bacteroidete *C. hutchinsonii*. The extracellular cellulase *Ch*Cel5A has been shown to release ~ 0.15 μM cellobiose and ~ 0.08 μM cellotriose from 3.3% (*w*/*v*) filter paper over 24 h [36]. In comparison, Cel5C_N generated ~ 800 μM of cellobiose from 1% Avicel in 24 h (Fig. 3b). Although these experiments are not directly comparable, it is worth noting that Avicel has a higher crystallinity index and, in theory, should be more difficult to degrade [37]. Since the enzyme concentration was not reported in reference [36], it is possible that it was lower than the 1 μM used here for Cel5C_N. Enzymes from the Ca. P. polyenzymogenes cluster also demonstrated CMCase activity that was more than 10-fold higher than *C. hutchinsonii* periplasmic endocellulases reported to be essential for cellulose utilization (*Ch*Cel5B: ~ 1000 U/μmol, *Ch*Cel9C: 600 U/μmol) [8, 38, 39]. Notably, the *C. hutchinsonii* values may be underestimated due to the fact that no progress curves were recorded and the activity was measured at a time point (30 min) that potentially was after completion of the reaction.

Differences in the modes of action between Cel5A-D were identified using assays with cellodextrins (Glc_(5/6)_) (Fig. 3c, Additional file 13: Figure S7). Cel5A slowly released Glc_(2)_ and Glc_(3)_ from Glc_(5)_ as well as Glc_(2)_, Glc_(3)_ and Glc_(4)_ from Glc_(6)_, which could indicate the presence of at least six subsites. Cel5B was equally slow on Glc_(5)_ but much faster when acting on Glc_(6)_, indicating a strong preference for long cello-oligosaccharides. Moreover, Cel5B was the only enzyme that converted Glc_(6)_ exclusively to Glc_(3)_, clearly indicating that the substrate affinities of the different subsites in this membrane-associated lipoprotein differ compared to the other GH5 cellulases (Fig. 3c). Cel5C_N and Cel5D were too active to show the stepwise degradation of Glc_(5)_ or Glc_(6)_ in the assayed time points, degrading both substrates to completion in 30 s. Both enzymes produced Glc_(2)_ and Glc_(3)_ from Glc_(5)_, whereas Glc_(6)_ was converted to a mixture dominated by Glc_(2)_ but also containing Glc_(3)_. The enzymes thus cleave Glc_(6)_ by binding the substrate to subsites − 3 to + 3, producing Glc_(3)_, and to − 4 to + 2 or − 2 to + 4, to produce Glc_(4)_ which is further degraded to Glc_(2)_. Cel5C_R gave the same results as Cel5C_N, and Cel5C_CR showed the same rate and pattern of degradation as Cel5A. Characterization of the GH3 β-glucosidase revealed exo-activity on cellodextrins (Additional file 14: Figure S8). Predicted as periplasmic and membrane-bound, the GH3 enzyme could further degrade imported cellodextrins released by the GH5 containing enzymes to glucose. A putative GH94 cellobiose phosphorylase was also located within the Ca. P. polyenzymogenes cellulase cluster and was expressed and purified, but we could not detect any activity with the assay conditions used.

Analysis of hemicellulose degradation by the GH5 proteins and their individual domains revealed activity specific for unsubstituted substrates containing β-1,4-linked glucose units in the backbone (Additional file 15: Figure S9, Additional file 10: Supplemental Text S1). The inability of the otherwise highly active Cel5C_N to degrade decorated hemicellulose substrates (e.g. xyloglucan) was supported by structural characterization studies, which resolved the structure of Cel5C_N to 1.57 Å (Fig. 3d, Additional file 16: Figure S10, Additional file 17: Table S6). The structure displays a (β/α)_8_-barrel fold, typical to GH5 enzymes [34], although a comparably narrower active-site cleft was observed, similar to that of GH5 enzymes that specifically accommodate linear polysaccharides, such as cellulose [40].

## Discussion

Here, we have defined the novel Ca. MH11 family and revealed key functional capabilities of its representatives, with particular focus on a fiber-associated uncultured *Bacteroidetes* genotype that is inherent to the cow rumen. Analysis of the Ca. P. polyenzymogenes genome (~ 90% complete), proteomic detection of key enzymes and metabolic pathways demonstrating in situ metabolism, and characterization of a selection of recombinant cellulases indicate that this genotype is specialized to degrade an array of polysaccharides to succinate and acetate. Ca. P. polyenzymogenes and other Ca. MH11 members stand apart from rumen *Bacteroidetes* species studied to date owing to their predicted secretion of large multi-modular cellulases via the *Bacteroidetes*-specific T9SS, known to be crucial in degradation of crystalline cellulose and chitin in soil bacteria [20, 23].

Biochemical studies of complex multi-domain CAZymes is notoriously difficult, but the few examples in literature show that such enzymes may be very powerful [24, 25]. While further work on the secreted multi-modular enzymes of Ca. P. polyenzymogenes and the other Ca. MH11 members is needed, the biochemical characterization of a gene cluster containing four GH5 cellulases, three with T9SS secretion tags carried out here, already provides evidence for the degradative potential of this novel yet uncultured *Bacteroidetes*. The enzymes showed activity on crystalline cellulose, releasing cellotriose and cellobiose in vitro, whereas cellodextrin assays demonstrated different binding modes and substrate preferences, which likely reflect different activities on crystalline cellulose. The most active cellulase domain belonged to the T9SS-linked (GH5-GH5) multi-domain Cel5C, which so far has proven difficult to express as a full-length protein with two active domains. Our studies revealed clear differences in activity towards CMC, Avicel and cellodextrins, and differences in hemicellulolytic activity, indicative of different functionalities in vivo. In this respect, it is important to note that the substrates used for enzyme characterization used in this and many other studies, differ from the co-polymeric plant material that Ca. P. polyenzymogenes meets in the rumen.

## Conclusion

In summary, our characterization of Ca. MH11 and Ca. P. polyenzymogenes has expanded the current view of ruminal polysaccharide conversion, further implicating *Bacteroidetes*-affiliated phylotypes as contributors via a non-classical mechanism that does not depend upon known cellobiohydrolase enzymes, cellulosomes, or PUL structures. Considering the metabolic features of Ca. P. polyenzymogenes in context with its proteomic detection and predicted numerical abundance [9], it is likely that this population constitutes one of the many important yet poorly understood *Bacteroidetes* affiliates that are critical for the host to sustain its herbivorous lifestyle.

## Methods

### Metagenomic datasets

Sequencing of several shotgun metagenomic datasets as well as assembly and binning of the AGa genome bin was performed in its entirety in a previous study [9] and is briefly summarized here: the assembly of 268 Gbp of HiSeq metagenomic sequence resulted in 179,092 scaffolds, of which the 65 largest ranged in size from 0.5 to 1.5 Mbp. Scaffold integrity was validated via two independent indicators of scaffold integrity: (i) level and uniformity of read depth in sub-regions and (ii) mate-pair support. A total of 26,042 scaffolds greater than 10 kbp were used to generate draft genomes, which were binned using tetranucleotide frequencies and read coverage. The completeness of AGa was calculated as a fraction of the number of identified and the number of expected core genes within the *Bacteroidales* order.

### Phylogenetic analyses

All concatenated ribosomal trees (Fig. 1) used finished and permanent draft genomes downloaded (September 2016) from IMG [41] to create reference datasets for 16 ribosomal proteins chosen as single-copy phylogenetic marker genes (*rpL2*, *rpL3*, *rpL4*, *rpL5*, *rpL6*, *rpL14*, *rpL15*, *rpL16*, *rpL18*, *rpL22*, *rpL24*, *rpS3*, *rpS8*, *rpS10*, *rpS17*, *rpS19*). Each individual protein dataset was aligned using MUSCLE 3.8.31 and then manually curated to remove end gaps [42]. A maximum likelihood phylogeny for the concatenated alignment was conducted using RAxML version 8.3.1 under the LG model of evolution with 100 bootstrap replicates [43] and visualized in iTOL [44]. For sequence-based comparison, average amino acid identity (AAI) and average nucleotide identity (ANI) values were calculated using the ANI and AAI calculators from the Kostas lab calculator (http://enve-omics.ce.gatech.edu/).

### Functional genomics

The AGa genome bin was functionally annotated with the Integrated Microbial Genomes Expert Review (IMG genome id: 2061766007) [41], and overall metabolic pathways were evaluated using KEGG metabolic maps [45]. CAZymes of different functional classes (glycoside hydrolases, carbohydrate-binding modules, carbohydrate esterases) were named in accordance with the CAZy nomenclature and identified using hidden Markov model (HMM) searches (HMMERv3.0) [46], with dbCAN-computed HMM representatives of each CAZy family [47]. The specific cutoff was set to Gathering Threshold (HMMER). Identification of T9SS components as well as CTDs was done using HMMER 3.0 with previously computed HMMs [21]. The TIGR04183 HMM from TIGRFAM was used to identify the T9SS-CTD.

### Metaproteomics

Samples used for metaproteomics experiments were collected from in situ nylon bags containing 5 g of air-dried switch grass or corn stover that were placed in the rumen of two cannulated cows (designated Y and Z). Rumen-incubated biomass and bulk rumen fluid were collected after 48 h using the protocol previously established [9]. All animal procedures were carried out under an animal care and use protocol (IUCAC #06081) approved by the Committee for Animal Care and Use of Animals at the University of Illinois.

Samples were snap-frozen in liquid nitrogen immediately after being retrieved from the rumen and transported to the laboratory where they were stored at − 80 °C until further processing. For protein extraction, the rumen-incubated plant material was squeezed to separate the liquid fraction from the solid biomass. An aliquot (~ 5 mL) of the liquid fraction from the rumen-incubated biomass and ~ 5 mL of the bulk rumen fluid were transferred to a 15-mL tube. Prior to centrifuging these samples for 20 min at 12000×*g* (4 °C), 50 μL of 100× SIGMAFAST protease inhibitor was added. Supernatants were transferred to a new 15-mL tube and are referred to hereafter as “secretome” and “control” (or simply as “rumen fluid”), respectively. An aliquot (~ 2 g) of the solid fraction from the rumen-incubated biomass was ground using a Biopulverizer (Biospec, Bartlesville, OK) and liquid nitrogen. Prior to centrifuging the ground samples for 10 min at 12000×*g* (4 °C), 3 mL of 1× SIGMAFAST protease inhibitor in 100 mM NH_4_HCO_3_ was added. Supernatants were transferred to a new 15-mL tube. Obtained pellets and supernatants are referred to as “Fiber attached” and “Loosely adherent”, respectively (e.g. Additional file 7: Table S4). Supernatants representing the secretome and the fiber adherent fraction were concentrated further to 500 μL, using an Amicon 3K MWCO filter (Millipore, Billerica, MA). Prior to loading 2 mL of the corresponding supernatant to the filter, 20 μL of 100× SIGMAFAST protease inhibitor were added. A buffer exchange to 100 mM NH_4_HCO_3_ was performed during the concentration step.

Protein concentrations were determined using the bicinchoninic acid (BCA) protein assay (ThermoFisher Pierce, Waltham, MA). Urea and dithiothreitol (DTT) were added to all samples to a final concentration of 8 M and 10 mM, respectively, and incubated at 60 °C for 30 min to denature and reduce proteins. Samples were diluted 8-fold with 100 mM NH_4_HCO_3_, before CaCl_2_ trypsin were added to a 1-mM final concentration and in a 1:50 trypsin to protein (*w*/*w*) ratio, respectively. To achieve protein digestion, samples were placed in a 37 °C shaking incubator at 235 rpm for 3 h. Subsequently, the samples were centrifuged at 5525×*g* for 15 min (4 °C), and the supernatant was subjected to C18 solid-phase extraction (SPE) cleanup on 1 mL/100 mg Discovery columns (Sigma Supelco, Bellefonte, PA) according to manufacturer’s instructions. Briefly, the columns were conditioned with 3 mL methanol followed by 2 mL of 0.1% trifluoroacetic acid (TFA). After the samples were loaded on the columns, they were rinsed with 4 mL of 95:5 water to acetonitrile with 0.1% TFA. The columns were allowed to dry, after which the samples were eluted with 1 mL of 80:20 acetonitrile to water with 0.1% TFA. The samples were concentrated using a Speed Vac (ThermoFisher Scientific, Waltham, MA) to between 50 and 150 μL, and a final BCA protein assay was performed to quantitate the peptide mass. The samples were diluted to 0.1 μg/μL in water for analysis by LC-MS/MS.

Samples from rumen-incubated plant material and the bulk rumen fluid of the two animals were separated into 12 fractions as previously described [48]. The obtained fractions were analyzed by reversed-phase LC-MS/MS using a Waters nanoACQUITY™ UPLC system (Millford, MA) coupled with an Orbitrap Velos mass spectrometer from ThermoFisher Scientific (San Jose, CA). Samples were loaded onto a trap column followed by separation on a C_18_ analytical column. The analytical column was packed in-house by pumping a slurry of 3-μm Jupiter C_18_ stationary phase (Phenomenex, Torrence, CA) into a 70-cm long, 75-μm ID fused silica capillary tubing (Polymicro Technologies Inc., Phoenix, AZ). The trap column (150 μm ID) of 5-cm length was similarly made with 3.6-μm Aries C_18_ particles. Mobile phases consisted of 0.1% (*v*/*v*) formic acid in water (A) and 0.1% (*v*/*v*) formic acid in acetonitrile (B). The peptide concentrations in the samples were ~ 0.1 μg/μL, and 6 μL were loaded onto the trap column via a 5-μL sample loop for 30 min at a flow rate of 3 μL per minute. After 30 min, the trap column was switched in-line with the analytical flow and the peptides were separated on the analytical column using a 110-min linear gradient from 99% A to 5% A at a flow rate of 0.3 μL per minute. Mass spectrometry acquisition was initiated 15 min after the sample was loaded onto the analytical column, and mass spectra were recorded for 100 min. After the gradient was completed, the column was washed with 100% B and then reconditioned with 99% A for 30 min.

The effluents from the LC column were ionized by electrospray ionization and their mass was analyzed with the Orbitrap Velos mass spectrometer operated in data-dependent mode. A voltage of 2.2 kV was applied at the liquid junction for electrospray ionization. The inlet capillary to transfer ions into the mass spectrometer was maintained at 350 °C for ion de-solvation. A primary survey scan was performed in the mass range of 400 to 2000 Da at a resolution of 60,000 (defined at *m/z* 200) and automatic gain control setting of 1e6 ions. The top 10 highest intensity ions from the survey scan were selected for fragmentation in the ion trap using a 2-Da isolation window and collisionally induced dissociation with normalized collision energy of 35%. Mass spectra were recorded for 100 min by repeating this process with a dynamic exclusion of previously selected ions for 60 s. The obtained MS/MS scans were subsequently analyzed using MaxQuant [49] version 1.6.0.13 and proteins quantified using the MaxLFQ [50] algorithm implemented in MaxQuant. Peptides were identified by searching the MS/MS datasets against the Ca. P. polyenzymogene-affiliated open reading frames (ORFs) recovered from the cow rumen metagenome [9] and annotated by the IMG/MER metagenomic pipeline [41] (Additional file 7: Table S4). The protein sequence database used for searching had common contaminants, such as human keratin and bovine serum albumin, appended. Tolerance levels for peptide identifications were 6 ppm and 0.5 Da for MS and MS/MS, respectively, and two missed cleavages of trypsin were allowed. Carbomidomethylation of cysteine residues was used as a fixed modification, while oxidation of methionines and protein N-terminal acetylation were used as variable modifications. All identifications were filtered in order to achieve a protein false discovery rate of 1% using the target-decoy strategy. The software platform Perseus version 1.6.0.7 [51] was used for downstream interpretation and quality filtration, including removal of decoy database hits, hits only identified by site and contaminants. Finally, at least one unique peptide per protein was required for a protein to be considered as valid.

### Heterologous expression and purification of genes

Genes were synthesized by GeneArt (Regensburg, Germany) without predicted signal peptides (SignalP 4.0, [52]) and T9SS CTDs and cloned into pNIC-CH (Addgene plasmid #26117, a generous gift from Opher Giliadi) using ligation-independent cloning (Additional file 18: Table S7). Transformants were verified by sequencing. *Escherichia coli* BL21 strains harbouring the plasmids were pre-cultured overnight in a Luria broth (LB) containing kanamycin (50 μg/mL) at 37 °C, and the overnight cultures were 100 times diluted into bottles containing 500 mL LB containing kanamycin (50 μg/mL), followed by incubation at 37 °C in an LEX Bioreactor (Harbinger, Pune 411038, India). Expression was induced by adding IPTG to a final concentration of 0.5 mM at OD_600_ 0.5–1.0, followed by incubation for 24 h at 22 °C. Cells were harvested by centrifugation (3000×*g*, 10 min) and resuspended in lysis buffer (50 mM Tris-HCl, pH 8.0, 200 mM NaCl, 5 mM imidazole, 0.1 mg/ml lysozyme) before 30-min incubation on ice. Cells were disrupted by pulsed sonication (25% amplitude, 4 × 30 s, 1 s pulses), and debris was removed by centrifugation (25,000*g*, 10 min). The supernatant was filtered with 0.22-μm syringe filters before loading on 5 mL HisTrap HP Ni Sepharose columns (GE Healthcare). Proteins were eluted with a linear gradient towards 50 mM Tris-HCl, pH 8.0, 200 mM NaCl, 500 mM imidazole. The eluted fractions were concentrated, and the buffer changed to 50 mM Tris-HCl pH 8.0, 0.2 M NaCl, using Sartorius Vivaspin concentrators with a 10-kDa cutoff. When needed, further purification was performed by gel filtration (HiLoad Superdex 75, GE Healthcare) in 50 mM Tris-HCl pH 8.0, 200 mM NaCl. Protein purity was analyzed by SDS-PAGE, and protein concentrations were estimated by measuring the A_280_ and using theoretical molar extinction coefficients.

Cel5A and its individual domains were insoluble when expressed in pNIC-CH. Therefore, expression was attempted by screening various soluble fusion partners using the Expresso Solubility kit from Lucigen (Middleton, WI, USA). Cel5A was soluble with the N-terminal his-tag control, whereas Cel5A_N and Cel5A_C were soluble with the maltose-binding protein fusion partner. These proteins were expressed as described in the manual provided by Lucigen, in 50 mL LB containing kanamycin (30 μg/mL), in Erlenmeyer flasks with 220 rpm vertical shaking using the recommended inducer concentrations. The maltose-binding protein was removed using TEV-protease (Sigma, T4455) and purified as described in the manual provided by Lucigen.

### Hemicellulose enzyme assays

Enzymatic activity was assayed on Megazyme (Bray, Ireland) polysaccharides: barley β-glucan (P-BGBL), lichenan (P-LICHN), Konjac glucomannan (P-GLCML), arabinoxylan (Megazyme, P-WAXYL), tamarind xyloglucan (P-XYGLN), Guar galactomannan (P-GGMMV), Carob galactomannan (P-GALML), and Carboxymethyl Pachyman (P-CMPAC). The substrates (0.5% *w/v*) were incubated with 1 μM enzyme in 20 mM citrate buffer, pH 5.5 at 40 °C for 1 h. Reducing sugars were measured against a glucose standard curve using a modified DNS assay [53], where 100 μL sample was mixed with 100 μL DNS reagent and boiled in covered 96-well plates. One hundred fifty microliters was transferred to a new 96-well plate, and the absorbance was measured at 540 nM.

### Quantification of enzyme activity on CMC and barley β-glucan

Approximate initial rates were determined on CMC for those GH5 enzymes that displayed sufficient activity. One percent (*w*/*v*) CMC (Sigma-Aldrich, St. Louis, MO, USA) was incubated with varying enzyme concentrations in 20 mM citrate buffer pH 5.5, at 40 °C with 1000 rpm vertical shaking in an Eppendorf Comfort Thermomixer. Product formation was measured using DNS as described above. One unit of enzyme activity was defined as the amount of enzyme releasing reducing sugars equivalent to 1 μmol of glucose per minute. Units were calculated at enzyme concentrations and time points where approximate initial rates (i.e. linear progress curves) were achieved and the enzyme dose-response was linear. For enzymes with too low activity on CMC, barley β-glucan (Megazyme) was used as a substrate.

### Avicel degradation assay

Avicel PH101 (Sigma, PH11365) (1% *w*/*v*) was incubated with 1 μM enzyme for 24 h in 20 mM citrate buffer, pH 5.5 at 40 °C with 1000 rpm vertical shaking. Samples were taken at various time points, and the reaction was stopped by addition of NaOH to a final concentration of 0.1 M. Products were analyzed and quantified against a standard curve of cello-oligosaccharides (Megazyme) by high-performance anion-exchange chromatography with pulsed amperometric detection (HPAEC-PAD), using a Dionex ICS-3000 with a CarboPac PA1 column with 0.1 M NaOH as the starting mobile phase and a flow rate of 0.25 ml/min. Oligosaccharides were eluted by a multi-step linear gradient going from 0.1 M NaOH to 0.1 M NaOH/0.1 M NaOAc in 10 min, to 0.1 M NaOH/0.14 M NaOAc in 4 min, to 0.1 M NaOH/0.3 M NaOAc in 1 min, and to 0.1 M NaOH/1.0 M NaOAc in 2 min, before column reconditioning by applying 0.1 M NaOH for 11 min.

### Degradation of cellodextrins

Cellodextrins (0.1 mg/ml) of various lengths provided by Sigma (Glc_2_) or Megazyme (Glc_3–6_) were incubated with 0.25 μM enzyme in 20 mM citrate buffer, pH 5.5, at 40 °C with 1000 rpm vertical shaking. Samples were taken at various time points, and the reaction was stopped by the addition of NaOH to a final concentration of 0.1 M. Products were analyzed by HPAEC-PAD as described above. For the characterization of the GH3, 1 μM enzyme was used, and the reaction time was 25 h. The GH94 cellobiose phosphorylase was assayed with 1 μM enzyme on 20 mM Glc_(2)_ and Glc_(3)_, in 50 mM sodium phosphate buffer pH 6.0 for 24 h at 40 °C with 1000 rpm vertical shaking, before stopping the reaction by addition of NaOH to a final concentration of 0.1 M NaOH. Products were analyzed by HPAEC-PAD as described above.

### Binding to cellulose

The binding of Cel5A_C to cellulose was assayed by incubating 0.08 mg/ml protein with 1% (*w*/*v*) Avicel at 22 °C with 1000 rpm vertical shaking in 20 mM citrate buffer, pH 5.5. Samples were taken at various time points and filtered using a 96-well filter plate (Millipore) operated by a Millipore vacuum manifold to remove the cellulose. The concentration of protein in the supernatant was determined by A_280_ using an Eppendorf biophotometer. Hen egg-white lysozyme (Sigma) was used as a non-binding control.

### Crystallization, diffraction data collection, and structure determination

Cel5C_N crystals were produced by screening hanging drop vapor diffusion (100 μL well volume, 1 μl protein + 1 μl well solution) using the Morpheus HT-96 crystallization screen (Molecular Dimensions). Crystals formed in several conditions, with the best crystals forming in 0.09 M NaF, 0.09 M NaBr, 0.09 M NaI, 0.1 M imidazole, MES monohydrate pH 6.5, 20% PEG 500 MME, 20% PEG 20 K. The enzyme concentration was 15 mg/mL in 20 mM Tris-HCl, 0.2 M NaCl. Crystals were briefly transferred to a cryoprotectant comprised of 80% well solution and 20% ethylene glycol, then flash frozen in liquid nitrogen. X-ray diffraction maxima were collected at the Life Sciences Collaborative Access Team (LS-CAT) ID-G beamline at the Advanced Photon Source at Argonne National Labs, Argonne, IL. Diffraction data were collected to 1.2 Å and integrated using iMOSFLM [54]. The data was scaled to 1.4 Å using SCALA [55, 56] and phased via molecular replacement using PHASER [57] with pdb 3PZT [58] as the search model, followed by Autobuild within PHENIX [59]. For the final rounds of manual model building, the X-ray data were re-processed in Xia2 [60] and the structure was completed using Coot and refinement in PHENIX in several rounds [59, 61].

## Additional files

Additional file 1: **Figure S1.** Two-way average nucleotide identity (blue) and average amino acid identity (red) values calculated for reference *Bacteroidetes* genomes and the AGa genome. (DOCX 386 kb)

Additional file 2: **Table S1.** Key metabolic enzymes annotated within the genome of Ca. P. polyenzymogenes. (DOCX 53 kb)

Additional file 3: **Table S2.** CAZyme profiles for the seven Ca. MH11 affiliated genomes. (XLSX 12 kb)

Additional file 4: **Figure S2.** Exemplar T9SS-secreted multi-modular CAZymes with predicted activities towards cellulosic and hemicellulosic substrates. (DOCX 399 kb)

Additional file 5: **Figure S3.** Exemplar GH5-containing gene clusters in Ca. MH11 genomes. (DOCX 145 kb)

Additional file 6: **Table S3.** Open reading frame coordinates and CAZyme annotation for UBA genomes affiliated to Ca. MH11. (XLSX 182 kb)

Additional file 7: **Table S4.** Ca. P. polyenzymogenes-affiliated ORFs detected via HTP metaproteomic analysis of rumen microbiome samples. (XLSX 28 kb)

Additional file 8: **Table S5.** Specific activities of the GH5 enzymes. (DOCX 14 kb)

Additional file 9: **Figure S4.** Multiple alignment of GH5 domains. (DOCX 39 kb)

Additional file 10: Supplemental text Does the C-terminal Cel5C domain contain a mutation in its active site?. (DOCX 20 kb)

Additional file 11: **Figure S5.** Comparison of individual Cel5C domains and the two domains combined. (DOCX 40 kb)

Additional file 12: **Figure S6.** Cel5A_C binding to Avicel cellulose. (DOCX 36 kb)

Additional file 13: **Figure S7.** Degradation of Glc(_5_) and Glc(_6_) by GH5 enzymes. (DOCX 540 kb)

Additional file 14: **Figure S8.** Degradation of cellodextrins by the GH3 β-glucosidase. (DOCX 259 kb)

Additional file 15: **Figure S9.** Hemicellulose activities of GH5 cellulases. (DOCX 474 kb)

Additional file 16: **Figure S10.** Crystal structure of Cel5C_N. (DOCX 1145 kb)

Additional file 17: **Table S6.** Data collection and refinement statistics for the crystal structure of Cel5C_N. (DOCX 14 kb)

Additional file 18: **Table S7.** Expression constructs used in this study. (DOCX 13 kb)

## Abbreviations

AAI: Average amino acid identify
ANI: Average nucleotide identity
CAZymes: Carbohydrate-active enzymes
CBM: Carbohydrate-binding module
CMC: Carboxymethyl-cellulose
CTD: Carboxy-terminal domain
EMP: Embden-Meyerhof-Parnas pathway
GH: Glycosyl hydrolase
OAA: Oxaloacetate
PEP: Phosphoenolpyruvate
PUL: Polysaccharide utilization loci
T9SS: Type IX secretion system
UBA: Uncultured Bacteria and Archaea database
