# Supplementary material for: “Candidatus Paraporphyromonas polyenzymogenes” encodes multi-modular cellulases linked to the type IX secretion system
Source: Microbiome. 2018 Mar 1;6:44. doi: 10.1186/s40168-018-0421-8 (PMC5831590; doi:10.1186/s40168-018-0421-8)
Supplement: Supplementary file 1 — Figure S1. Two-way average nucleotide identity (blue) and average amino acid identity (red) values calculated for reference Bacteroidetes genomes and the AGa genome. (DOCX 386 kb) [file 40168_2018_421_MOESM1_ESM.docx]

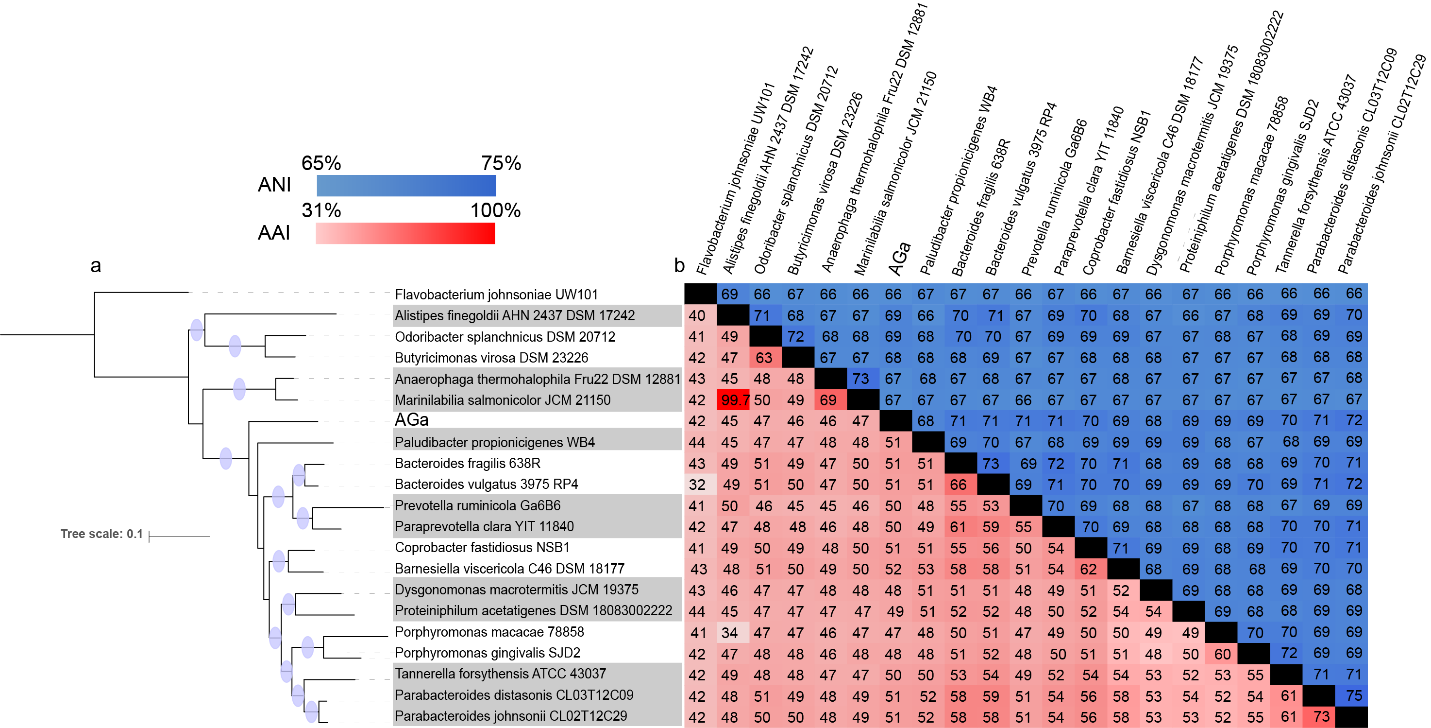


**Figure S1.** Two-way average nucleotide identity (blue) and average amino acid identity (red) values calculated for reference *Bacteroidetes* genomes and the AGa genome. (a) Concatenated ribosomal tree using 16 single copy ribosomal proteins. Purple circles indicate bootstrap support >70. Gray and white boxes indicate separate families according to Ormerod *et al.* 2016 [1] and are presented from top to bottom as follows: *Flavobetacteriaceae*, *Rikenellaceae*, *Marinifilaceae*, *Marinilabilaceae*, AGa, *Paludibacteraceae*, *Bacteroidaceae*, *Prevotellaceae*, *Barnesiellaceae*, *Dysgonamonadaceae*, *Porphyromonadaceae*, *Tannerellaceae*. (b) Pairwise ANI (blue) and AAI (red) values calculated using the algorithm described by Goris et al. 2007 [2] with color scaled by percent identity.

1. Ormerod KL, Wood DLA, Lachner N, Gellatly SL, Daly JN, Parsons JD, Dal'Molin CGO, Palfreyman RW, Nielsen LK, Cooper MA *et al*: **Genomic characterization of the uncultured Bacteroidales family S24-7 inhabiting the guts of homeothermic animals.** *Microbiome* 2016, **4**:36.

2. Goris J, Konstantinidis KT, Klappenbach JA, Coenye T, Vandamme P, Tiedje JM: **DNA-DNA hybridization values and their relationship to whole-genome sequence similarities.** *Int J Syst Evol Microbiol* 2007, **57**:81-91.
