# Supplementary material for: “Candidatus Paraporphyromonas polyenzymogenes” encodes multi-modular cellulases linked to the type IX secretion system
Source: Microbiome. 2018 Mar 1;6:44. doi: 10.1186/s40168-018-0421-8 (PMC5831590; doi:10.1186/s40168-018-0421-8)
Supplement: Supplementary file 2 — Table S1. Key metabolic enzymes annotated within the genome of Ca. P. polyenzymogenes. (DOCX 53 kb) [file 40168_2018_421_MOESM2_ESM.docx]

**Table S1.** Key metabolic enzymes annotated within the genome of *Ca.* P. polyenzymogenes. E.C., Pfam, CAZy family and TIGRfam numbers (where available) as well as gene names and IMG gene ID numbers are provided. The TIGR04183 HMM from TIGRFAM was used to identify T9SS-CTD. Rows highlighted in green indicate ORFs that were detected in metaproteomic analysis in two replicate animals, rows highlighted in yellow indicate detection in one animal only (**Figure 2**, **Additional file 7: Table S4**).

| Protein/Function | Annotation (EC/pfam/TIGRfam) | Gene/CAZy | Gene IMG ID | T9SS-CTD |
| --- | --- | --- | --- | --- |
| Sugar utilization |  |  |  |  |
| mannose-1-phosphate guanylyltransferase | 2.7.7.22 | GMPP/MPI | _HiSeq_18092740 |  |
| β-1,4-mannooligosaccharide phosphorylase | 2.4.1.319 | GH130 | nd |  |
| 4-O-beta-D-mannosyl-D-glucose phosphorylase | 2.4.1.281 | GH130 | nd |  |
| mannose-6-phosphate isomerase | 5.3.1.8 | MPI | _HiSeq_18012640 |  |
| phosphomannomutase | 5.4.2.8 | PMM | _HiSeq_18094410 |  |
| Sugar kinases, ribokinase family | 2.7.1.4 |  | _HiSeq_15063060 |  |
| alpha-glucan phosphorylases | 2.4.1.1 | glgP | _HiSeq_18012570 |  |
| phosphoglucomutase | 5.4.2.2 | pgm | _HiSeq_18014350 |  |
| Polysaccharide utilization |  |  |  |  |
| β-glucosidase | 3.2.1.21 | GH3 | _HiSeq_18101970 |  |
| - | - | GH3 | _HiSeq_23331270 |  |
| β-galactosidase; β-mannosidase; β-glucuronidase | 3.2.1.23; 3.2.1.25; 3.2.1.31 | GH2 | _HiSeq_18101690 | + |
| cellobiose phosphorylase | 2.4.1.20 | GH94 | _HiSeq_05875360 |  |
| - | - | GH94 | _HiSeq_23331290 |  |
| endo-β-1,4-glucanase; endo-β-1,4-xylanase; licheninase; | 3.2.1.4; 3.2.1.8; | GH5+CBM6 | _HiSeq_05874540 | + |
| mannan endo-β-1,4-mannosidase; xyloglucanase | 3.2.1.73; 3.2.1.78; | GH5+GH26 | _HiSeq_05875190 | + |
| - | 3.2.1.151 | GH5+CBM6 | _HiSeq_05875280 | + |
| - | - | GH5 | _HiSeq_14128290 |  |
| - | - | GH5+CBM35 | _HiSeq_17667960 | + |
| - | - | GH5 | _HiSeq_18099380 |  |
| - | - | GH5 | _HiSeq_18100670 |  |
| - | - | GH5 | _HiSeq_18102810 |  |
| - | - | GH5+CBM6 | _HiSeq_23331250 | + |
| - | - | GH5 | _HiSeq_23331280 |  |
| - | - | GH5+GH5 | _HiSeq_23331320 | + |
| - | - | GH5 | _HiSeq_23331330 | + |
| - | - | GH5+GH5 | _HiSeq_23331410 | + |
| endo-β-1,4-glucanase; cellobiohydrolase; licheninase; | 3.2.1.4; 3.2.1.91; | GH9 | _HiSeq_15059240 | + |
| β-glucosidase; xyloglucanase | 3.2.1.6; 3.2.1.21; | GH9 | _HiSeq_18091690 | + |
| - | 3.2.1.151 | GH9+CBM30 | _HiSeq_18094320 |  |
| - | - | GH9 | _HiSeq_23696780 | + |
| - | - | GH74x3 | _HiSeq_18102130 | + |
| endo-β-1,4-xylanase | 3.2.1.8 | GH10+CBM6 | _HiSeq_15059270 |  |
| - | - | GH10+CBM6 | _HiSeq_18101540 | + |
| - | - | GH11+CBM6 | _HiSeq_05874250 |  |
| - | - | GH11 | _HiSeq_05874280 |  |
| - | - | GH11+CBM6 | _HiSeq_05874300 |  |
| - | - | GH11+CE4 | _HiSeq_15061270 | + |
| - | - | GH11+GH8 +CBM6 | _HiSeq_15061710 | + |
| - | - | GH11 | _HiSeq_18100680 | + |
| β-xylosidase; α-L-arabinofuranosidase; arabinanase | 3.2.1.37; 3.2.1.55; | GH43+CE1 +CBM6x2 | _HiSeq_05874410 | + |
| - | 3.2.1.99 | GH43+CBM6x2 | _HiSeq_05874570 | + |
| - | - | GH43+CBM6x2 | _HiSeq_15059850 | + |
| - | - | GH43+CBM6x2 | _HiSeq_15059880 | + |
| - | - | GH43+CBM13x2 | _HiSeq_17668040 |  |
| - | - | GH43+ GH30 +CBM13 | _HiSeq_18013240 | + |
| β-xylosidase | 3.2.1.37 | GH39+CBM6 | _HiSeq_15062000 |  |
| - | - | GH39+CBM6 | _HiSeq_18100720 |  |
| endo-1,3-β-glucanase; endo-1,3(4)-β-glucanase; licheninase; xyloglucanase | 3.2.1.39;3.2.1.6; 3.2.1.73; 3.2.1.151 | GH16  GH16+CBM13 | _HiSeq_13171140  _HiSeq_18013230 | + |
| β-glucosylceramidase; β-1,6-glucanase; β-xylosidase | 3.2.1.45; 3.2.1.75; | GH30+CBM44 +CBM6 | _HiSeq_05875040 | + |
| - | 3.2.1.37 | GH30 | _HiSeq_13171090 | + |
| endo-β-1,4-galactanase | 3.2.1.89 | GH53 | _HiSeq_15061280 |  |
| α-amylase; pullulanase | 3.2.1.1; 3.2.1.41 | GH13 | _HiSeq_18092110 |  |
|  |  | GH13+CBM48 | _HiSeq_18094020 |  |
|  |  | GH57 | _HiSeq_15059740 |  |
| amylo-α-1,6-glucosidase | 3.2.1.33 | GH133 | _HiSeq_15059720 |  |
| α-glucosidase; α-galactosidase; glucoamylase | 3.2.1.20; 3.2.1.22; | GH97 | _HiSeq_02681430 |  |
|  | 3.2.1.3 |  |  |  |
| amylomaltase or 4-α-glucanotransferase | 2.4.1.25 | GH77+CBM20x2 | _HiSeq_25046450 |  |
|  |  |  |  |  |
| α-N-acetylgalactosaminidase | 3.2.1.49 | GH109 | _HiSeq_18098610 |  |
|  |  | GH109 | _HiSeq_15060990 |  |
| α-glucosidase; α-galactosidase; α-glucuronidase; α-galacturonase | 3.2.1.20; 3.2.1.22; | GH4 | _HiSeq_18101800 |  |
|  | 3.2.1.139; |  | _HiSeq_25048130 |  |
|  | 3.2.1.67 |  |  |  |
| chitinase; lysozyme | 3.2.1.14; 3.2.1.17 | GH19 | _HiSeq_23696840 |  |
|  |  | GH23+CBM50 | _HiSeq_18013720 |  |
|  |  | GH23 | _HiSeq_18094370 |  |
|  |  | GH23 | _HiSeq_18100420 |  |
| Lysozyme; endo-β-N-acetylglucosaminidase | 3.2.1.17; 3.2.1.- | GH73+CBM50x2 | _HiSeq_15060660 |  |
|  |  | GH73+CBM50 | _HiSeq_17668010 |  |
| α-L-arabinofuranosidase | 3.2.1.55 | GH51+CBM4 | _HiSeq_05875420 |  |
| acetyl xylan esterase | 3.1.1.1.72 | CE1 | _HiSeq_05875270 |  |
| - | - | CE1 | _HiSeq_14128560 |  |
| - | - | CE1+CBM6 | _HiSeq_15059860 | + |
| - | - | CE1+CBM6 | _HiSeq_15059870 | + |
| - | - | CE1 | _HiSeq_18013360 | + |
| - | - | CE1 | _HiSeq_18101480 |  |
| - | - | CE1+CBM48 | _HiSeq_25047260 |  |
| - | - | CE2 | _HiSeq_18012920 |  |
| - | - | CE3 | _HiSeq_14128700 |  |
| - | - | CE3 | _HiSeq_18013020 |  |
| - | - | CE3 | _HiSeq_25047240 | + |
| acetyl xylan esterase; chitin deacetylase | 3.1.1.1.72; | CE4 | _HiSeq_18100780 |  |
| - | 3.5.1.41 | CE4 | _HiSeq_15063010 |  |
| - | - | CE4 | _HiSeq_18012680 |  |
| acetyl xylan esterase | 3.1.1.1.72 | CE6 | _HiSeq_05874300 |  |
| - | - | CE6 | _HiSeq_15060880 | + |
| - | - | CE6+CBM6 | _HiSeq_18093420 |  |
| pectin methylesterase | 3.1.1.11 | CE8 | _HiSeq_13171100 |  |
|  |  | CE15 | _HiSeq_05874260 | + |
|  |  | CE15 | _HiSeq_05874270 | + |
| inulin-binding; cellulose and xyloglucan | na | CBM38+CBM44 | _HiSeq_18099440 |  |
| sialic acid binding | na | CBM40 | _HiSeq_18099060 | + |
| cellulose and xyloglucan |  | CBM44 | _HiSeq_05874700 | + |
| - |  | CBM44 | _HiSeq_18012870 |  |
| chitin binding | na | CBM50x4 | _HiSeq_18099180 |  |
| - | na | CBM50x2 | _HiSeq_22511140 |  |
| - |  | CBM50 | _HiSeq_25048030 |  |
| β-1,3-glucan binding |  | CBM56 | _HiSeq_18091650 | + |
| - |  | CBM56 | _HiSeq_18093990 | + |
| - |  | CBM56 | _HiSeq_18099540 |  |
| - |  | CBM56 | _HiSeq_22510790 | + |
| amorphous cellulose; β-1,4-xylan; β-1,3-glucan: β-1,3-1,4-glucan; β-1,4-glucan. | na | CBM6 | _HiSeq_18101700 |  |
| β-1,4-galactan binding | na | CBM61 | _HiSeq_18100360 | + |
| Cellulose binding | na | CBM63 | _HiSeq_18093120 |  |
| L-rhamnose binding | na | CBM67 | _HiSeq_15060050 | + |
| - | na | CBM67 | _HiSeq_18094700 | + |
| Starch binding | na | CBM69 | _HiSeq_18102360 |  |
|  |  | CBM69 | _HiSeq_13171100 |  |
|  |  | PL11 | _HiSeq_15062970 | + |
|  |  | PL14 | _HiSeq_15063140 | + |
|  |  | PL9 | _HiSeq_18101700 |  |
|  |  |  |  |  |
| Glycolysis/Gluconeogenesis |  |  |  |  |
| glucokinase | 2.7.1.2 | glk | _HiSeq_15058690 |  |
| glucose-6-phosphate isomerase | 5.3.1.9 | pgi | _HiSeq_18012830 |  |
| 6-phosphofructokinase | 2.7.1.11 | pfkA | _HiSeq_14128710 |  |
| pyrophosphate-dependent phosphofructokinase | 2.7.1.90 | pfk | _HiSeq_18095210 |  |
| fructose-bisphosphate aldolase | 4.1.2.13 | fba | _HiSeq_23331400 |  |
| triosephosphate isomerase | 5.3.1.1 | tpiA | _HiSeq_13171240 |  |
| glyceraldehyde-3-phosphate dehydrogenase | 1.2.1.59 | gap | _HiSeq_18014140 |  |
| phosphoglycerate mutase | 5.4.2.11 | gpm | _HiSeq_18092340 |  |
| phosphoglycerate kinase | 2.7.2.3 | pgk | _HiSeq_18013350 |  |
| enolase | 4.2.1.11 | eno | _HiSeq_17667970 |  |
| pyruvate kinase | 2.7.1.40 | pyk | nd |  |
| pyruvate phosphate dikinase | pfam01326 | PpdK | _HiSeq_15057570 |  |
| pyruvate:ferredoxin (flavodoxin) oxidoreductase, homodimeric | 1.2.7.- | por | _HiSeq_18104410 |  |
| Pyruvate:ferredoxin oxidoreductase and related 2-oxoacid:ferredoxin oxidoreductases, alpha subunit | 1.2.7.1 | porA | _HiSeq_05874380 |  |
| Pyruvate:ferredoxin oxidoreductase and related 2-oxoacid:ferredoxin oxidoreductases, beta subunit | 1.2.7.1 | porB | _HiSeq_05874400 |  |
| pyruvate dehydrogenase E2 component | 2.3.1.12 | DLAT | nd |  |
| pyruvate formate-lyase | 2.3.1.54 | pflD | nd |  |
| oxaloacetate decarboxylase, alpha subunit | 4.1.1.3 | oadA | _HiSeq_18099580 |  |
| Na+-transporting oxaloacetate decarboxylase beta subunit | 4.1.1.3 | oadB | _HiSeq_18099570 |  |
| Malate dehydrogenase (oxaloacetate-decarboxylating) (NADP(+)) | 1.1.1.40 | maeB | _HiSeq_18013980 |  |
| phosphotransacetylase | 2.3.1.8 | pta | _HiSeq_15061050 |  |
| acetate kinase | 2.7.2.1 | ackA | _HiSeq_15061060 |  |
| phosphoenolpyruvate carboxykinase (GTP) | 4.1.1.32 | pckA | _HiSeq_25046800 |  |
| phosphoenolpyruvate carboxykinase | 4.1.1.32 | pckA | _HiSeq_25046520 |  |
| phosphoenolpyruvate carboxykinase | 4.1.1.49 | pckA | _HiSeq_05874790 |  |
| Pyruvate/oxaloacetate carboxyltransferase | 6.4.1.1 | PC | _HiSeq_18099580 |  |
| Malate/lactate dehydrogenases | 1.1.1.37 | mdh | _HiSeq_18101800 |  |
| fumarase | 4.2.1.2 | fumA | _HiSeq_23696760 |  |
| Outer membrane protein and related peptidoglycan-associated (lipo)proteins |  |  | _HiSeq_15062640 |  |
| succinate dehydrogenase subunit B | 1.3.5.4 | frdB | _HiSeq_15062650 |  |
| succinate dehydrogenase subunit A | 1.3.5.4 | frdA | _HiSeq_15062660 |  |
| succinate dehydrogenase subunit C | 1.3.5.4 | frdC | _HiSeq_15062670 |  |
| Pentose phosphate pathway (PPP) |  |  |  |  |
| xylose isomerase | 5.3.1.5 |  |  |  |
| xylulokinase | 2.7.1.17 | xylB |  |  |
| ribulose-phosphate 3-epimerase | 5.1.3.1 | rpe | _HiSeq_15057760 |  |
| ribose-5-phosphate isomerase | 5.3.1.6 | rpiA | _HiSeq_18102670 |  |
| transketolase | 2.2.1.1 | tkt | _HiSeq_18092390 |  |
| transaldolase | 2.2.1.2 | tal | - |  |
| Sugar kinases, ribokinase family | 2.7.1.15 | RBKS | _HiSeq_18013180 |  |
| ribose-phosphate pyrophosphokinase | 2.7.6.1 | PRPS | _HiSeq_18013000 |  |
| Fatty acid metabolism |  |  |  |  |
| biotin carboxylase | 6.3.4.14 | BCCP | _HiSeq_18101910 |  |
| biotin carboxylase | 6.3.4.14 | BCCP | _HiSeq_15057000 |  |
| biotin carboxylase | 6.3.4.14 | BCCP | _HiSeq_18101330 |  |
| biotin carboxyl carrier protein | pfamF00364 | BC | _HiSeq_18101920 |  |
| Acetyl-CoA carboxylase, carboxyltransferase component (subunits alpha and beta) | 6.4.1.2 | accA | _HiSeq_18101930 |  |
| 3-oxoacyl-(acyl-carrier-protein) synthase | pfam00109 | KAS | _HiSeq_23331840 |  |
| 3-oxoacyl-(acyl-carrier-protein) synthase | pfam13723 | KAS | _HiSeq_23331830 |  |
| 3-oxoacyl-(acyl-carrier-protein) synthase |  | KAS | _HiSeq_23331900 |  |
| [Acyl-carrier-protein] S-malonyltransferase | 2.3.1.39 | fabD | _HiSeq_14128860 |  |
| 3-oxoacyl-(acyl-carrier-protein) synthase III | 2.3.1.180 | fabH | _HiSeq_14128930 |  |
| enoyl-(acyl-carrier-protein) reductase II | 1.3.1.9 | fabK | _HiSeq_14128940 |  |
| 3-oxoacyl-[acyl-carrier-protein] synthase II | 2.3.1.179 | fabF | _HiSeq_14128950 |  |
| 3-oxoacyl-(acyl-carrier-protein) synthase | 2.3.1.41 | fabB | _HiSeq_18093500 |  |
| 3-oxoacyl-[acyl-carrier-protein] reductase | 1.1.1.100 | fabG | _HiSeq_14129100 |  |
| Long-chain acyl-CoA synthetases (AMP-forming) | 6.2.1.3 | ACSL | _HiSeq_15057360 |  |
| Long-chain acyl-CoA synthetases (AMP-forming) | 6.2.1.3 | ACSL | _HiSeq_15057370 |  |
| Aspartate and glutamate metabolism |  |  |  |  |
| glutamate synthase (NADH) small subunit | 1.4.1.14 | GltBD | _HiSeq_18095680 |  |
| glutamate synthase (NADH) large subunit | 1.4.1.14 | GltBD | _HiSeq_18095690 |  |
| asparagine synthase (glutamine-hydrolyzing) | 6.3.5.4 | AsnB | _HiSeq_18095670 |  |
| carbamoyl-phosphate synthase large subunit | 6.3.5.5 | CAD | _HiSeq_14128240 |  |
| carbamoyl-phosphate synthase large subunit | 6.3.5.5 | CAD | _HiSeq_15063130 |  |
| Glutamine phosphoribosylpyrophosphate amidotransferase | 2.4.2.14 | purF | _HiSeq_14128260 |  |
| glutamine--fructose-6-phosphate transaminase | 2.6.1.16 | glmS | _HiSeq_18095340 |  |
| aspartate semialdehyde dehydrogenase | 1.2.1.11 | asd | _HiSeq_05100660 |  |
| aspartate-ammonia ligase | 6.3.1.1 | AsnA | _HiSeq_18093680 |  |
| aspartate carbamoyltransferase | 2.1.3.2 | pyrB | _HiSeq_05100550 |  |
| argininosuccinate synthase | 6.3.4.5 | argG | _HiSeq_25047950 |  |
| argininosuccinate lyase | 4.3.2.1 | argH | _HiSeq_23696820 |  |
| Adenylosuccinate synthetase | 6.3.4.4 | purA | _HiSeq_18100380 |  |
| Adenylosuccinate lyase | 4.3.2.2 | purB | _HiSeq_18014660 |  |
| L-aspartate oxidase | 1.4.3.16 | AO | _HiSeq_15059520 |  |
| Histidinol-phosphate/aromatic aminotransferase and cobyric acid decarboxylase | 2.6.1.1 | GOT | _HiSeq_18098640 |  |
| Aspartate/tyrosine/aromatic aminotransferase | 2.6.1.1 | GOT | _HiSeq_18092230 |  |
| Aspartate/tyrosine/aromatic aminotransferase | 2.6.1.1 | GOT | _HiSeq_18093980 |  |
| Aspartate/tyrosine/aromatic aminotransferase | 2.6.1.1 | GOT | _HiSeq_18094970 |  |
| Aspartokinases | 2.7.2.4 | lysC | _HiSeq_18094870 |  |
| Nitrogen metabolism |  |  |  |  |
| nitrogen regulatory protein P-II | pfam00543 | PII | _HiSeq_15060020 |  |
| ammonium transporter | pfam00909 | AmtB | _HiSeq_15060030 |  |
| Glutamate synthase (NADPH) | 1.4.1.13 | GLT | _HiSeq_18014540 |  |
| NAD(P)H-flavin reductase |  |  | _HiSeq_18014550 |  |
| glutamate synthase (NADH) small subunit | 1.4.1.14 | GltBD | _HiSeq_18095680 |  |
| glutamate synthase (NADH) large subunit | 1.4.1.14 | GltBD | _HiSeq_18095690 |  |
| asparagine synthase (glutamine-hydrolyzing) | 6.3.5.4 | AsnB | _HiSeq_18095670 |  |
| glutamate dehydrogenase (NAD/NADP) | 1.4.1.3 | gdhA | _HiSeq_02681420 |  |
| glutamate dehydrogenase [NAD(P)+] | 1.4.1.4 | gdhA | _HiSeq_22511110 |  |
| L-glutamine synthetase | 6.3.1.2 | glnA | _HiSeq_18098300 |  |
| L-glutamine synthetase | 6.3.1.2 | glnA | _HiSeq_18102870 |  |
| aspartate-ammonia ligase | 6.3.1.1 | AsnA | _HiSeq_18093680 |  |
| Histidine ammonia-lyase | 4.3.1.3 | HAL | _HiSeq_23696710 |  |
| Dinitrogenase iron-molybdenum cofactor. | pfam02579 | NifX | _HiSeq_14128380 |  |
| Mo-nitrogenase iron protein subunit | 1.18.6.1 | NifH | _HiSeq_14128390 |  |
| Dinitrogenase iron-molybdenum cofactor. | pfam02579 |  | _HiSeq_14128400 |  |
| Nitrogenase molybdenum-iron protein, alpha and beta chains | pfam00148 |  | _HiSeq_14128410 |  |
| Nitrogenase molybdenum-iron protein, alpha and beta chains | pfam00148 |  | _HiSeq_14128420 |  |
| Oxidative phosphorylation/Energy metabolism |  |  |  |  |
| pyrophosphatase | 3.6.1.1 | ppa |  |  |
| NADH dehydrogenase subunit A | 1.6.5.3 | nuoA | _HiSeq_18094860 |  |
| NADH dehydrogenase subunit B | 1.6.5.3 | nuoB | _HiSeq_18094850 |  |
| NADH dehydrogenase subunit C/D | 1.6.5.3 | nuoC/D | _HiSeq_18094840 |  |
| NADH-quinone oxidoreductase subunit H | 1.6.5.3 | nuoH | _HiSeq_18094830 |  |
| NADH-quinone oxidoreductase subunit I | 1.6.5.3 | nuoI | _HiSeq_18094820 |  |
| NADH-quinone oxidoreductase subunit J | 1.6.5.3 | nuoJ | _HiSeq_18094810 |  |
| NADH dehydrogenase subunit K | 1.6.5.3 | nuoK | _HiSeq_18094800 |  |
| NADH dehydrogenase subunit L | 1.6.5.3 | nuoL | _HiSeq_18094790 |  |
| NADH dehydrogenase subunit M | 1.6.5.3 | nuoM | _HiSeq_18094780 |  |
| NADH dehydrogenase subunit N | 1.6.5.3 | nuoN | _HiSeq_18094770 |  |
| NADH:ubiquinone oxidoreductase, Na(+)-translocating, A subunit | 1.6.5.- | nqrA | _HiSeq_18095070 |  |
| NADH:ubiquinone oxidoreductase, Na(+)-translocating, B subunit | 1.6.5.- | nqrB | _HiSeq_18095080 |  |
| NADH:ubiquinone oxidoreductase, Na(+)-translocating, C subunit | 1.6.5.- | nqrC | _HiSeq_18095090 |  |
| NADH:ubiquinone oxidoreductase, Na(+)-translocating, D subunit | 1.6.5.- | nqrD | _HiSeq_18095100 |  |
| NADH:ubiquinone oxidoreductase, Na(+)-translocating, E subunit | 1.6.5.- | nqrE | _HiSeq_18095110 |  |
| NADH:ubiquinone oxidoreductase, Na(+)-translocating, F subunit | 1.6.5.- | nqrF | _HiSeq_18095120 |  |
| ATP synthase F1 subcomplex alpha subunit | 3.6.3.14 | ATPF1A | _HiSeq_18094520 |  |
| ATP synthase F1 subcomplex beta subunit | 3.6.3.14 | ATPF1B | _HiSeq_18094450 |  |
| ATP synthase F1 subcomplex gamma subunit |  | ATPF1G | _HiSeq_18094530 |  |
| ATP synthase F1 subcomplex delta subunit |  | ATPF1D | _HiSeq_18094510 |  |
| ATP synthase F1 subcomplex epsilon subunit |  | ATPF1E | _HiSeq_18094460 |  |
| ATP synthase F0 subcomplex A subunit |  | ATPF0A | _HiSeq_18094480 |  |
| ATP synthase F0 subcomplex B subunit |  | ATPF0B | _HiSeq_18094500 |  |
| ATP synthase F0 subcomplex C subunit |  | ATPF0C | _HiSeq_18094490 |  |
| Transporters |  |  |  |  |
| magnesium Mg(2+) and cobalt Co(2+) transport protein | pfam01544 | corA | _HiSeq_18094080 |  |
| amino acid/peptide transporter (Peptide:H+ symporter) | pfam00854 |  | _HiSeq_18094270 |  |
| TonB family C-terminal domain | pfam03544 |  | _HiSeq_18102430 |  |
| sodium/proton antiporter, NhaD family | TC 2.A.62 |  | _HiSeq_18102440 |  |
| Na+/phosphate symporter | pfam02690 |  | _HiSeq_18102780 |  |
| TonB family C-terminal domain | pfam03544 |  | _HiSeq_05100300 |  |
| Na+-dependent transporter | pfam00209 | SNF | _HiSeq_05100320 |  |
| outer membrane transport energization protein | TC 2.C.1.1.1 | ExbB | _HiSeq_23696210 |  |
| Biopolymer transport protein | pfam02472 | ExbD | _HiSeq_23696220 |  |
| TonB family C-terminal domain | pfam03544 |  | _HiSeq_23696230 |  |
| ABC-type nitrate/sulfonate/bicarbonate transport systems, periplasmic components | pfam09084 |  | _HiSeq_18102960 |  |
| ABC-type nitrate/sulfonate/bicarbonate transport system, permease component | pfam00528 |  | _HiSeq_18102970 |  |
| ABC-type nitrate/sulfonate/bicarbonate transport system, ATPase component | pfam00005 |  | _HiSeq_18102980 |  |
| protein translocase subunit secY/sec61 alpha | pfam00344 | secY | _HiSeq_15058040 |  |
| protein translocase subunit secA | pfam07517 | secA | _HiSeq_18014380 |  |
| protein translocase subunit secF/protein translocase subunit secD | pfam02355 | secD_secF | _HiSeq_14129120 |  |
| Stress response |  |  |  |  |
| Superoxide dismutase [Fe] | 1.15.1.1 | sodB | _HiSeq_25046980 |  |
| Rubrerythrin | pfam02915 | Rth | _HiSeq_18099220 |  |
| Rubrerythrin | pfam02915 | Rth | _HiSeq_15062310 |  |
| Rubrerythrin | pfam02915 | Rth | _HiSeq_13171030 |  |
| Thioredoxin reductase | 1.8.1.9 | TrxR | _HiSeq_18099120 |  |
| Peroxiredoxin | 1.11.1.15 | Bcp | _HiSeq_18098360 |  |
| Type IX secretion system |  |  |  |  |
| porQ |  | porQ | _HiSeq_14128740 |  |
| porU |  | porU | _HiSeq_18092490 |  |
| porV |  | porV | _HiSeq_18102920 |  |
| gld A (transmembrane ATP-binding-cassette transporter) |  | gldA | _HiSeq_25046410 |  |
| gld F (transmembrane ATP-binding-cassette transporter) |  | gldF | _HiSeq_18099500 |  |
| gld G (transmembrane ATP-binding-cassette transporter) |  | gldG | _HiSeq_23331550 |  |
| gld H (lipoprotein of unknown function) |  | gldH | _HiSeq_25046480 |  |
| gld J (lipoprotein of unknown function) | TIGR03524 | gldJ | _HiSeq_15059580 |  |
| gldK | TIGR03525 | gldK | _HiSeq_15058810 |  |
| gld L (Uncharacterized membrane protein) | TIGR03513 | gldL | _HiSeq_15058800 |  |
| gld M | TIGR03517 | gldM | _HiSeq_15058790 |  |
| gld N (periplasmic protein) | TIGR03523 | gldN | _HiSeq_15058780 |  |
| sprA-3 | TIGR04189 | Sov | _HiSeq_15059650 |  |
| sprE |  | porW | _HiSeq_15059770 |  |
| sprT |  | sprT | _HiSeq_15060270 |  |
|  |  |  |  |  |
