# Supplementary material for: “Candidatus Paraporphyromonas polyenzymogenes” encodes multi-modular cellulases linked to the type IX secretion system
Source: Microbiome. 2018 Mar 1;6:44. doi: 10.1186/s40168-018-0421-8 (PMC5831590; doi:10.1186/s40168-018-0421-8)
Supplement: Supplementary file 5 — Figure S3. Exemplar GH5-containing gene clusters in Ca. MH11 genomes. (DOCX 145 kb) [file 40168_2018_421_MOESM5_ESM.docx]

**
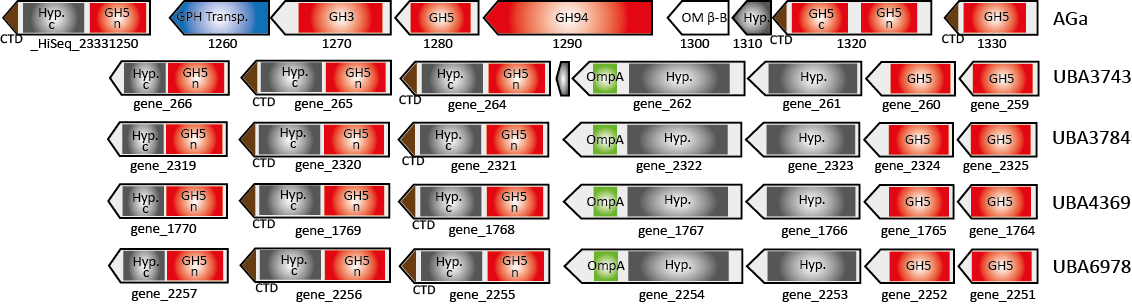
**

**Figure S3.** Exemplar GH5-containing gene clusters in *Ca.* MH11 genomes. CTD denotes a carboxy-terminal domain that infers export via T9SS. Hyp indicates domains with no known function. ORF coordinates and dbCAN annotations for UBA genomes are found in **Additional file 6: Table S3**.
