# Supplementary material for: “Candidatus Paraporphyromonas polyenzymogenes” encodes multi-modular cellulases linked to the type IX secretion system
Source: Microbiome. 2018 Mar 1;6:44. doi: 10.1186/s40168-018-0421-8 (PMC5831590; doi:10.1186/s40168-018-0421-8)
Supplement: Supplementary file 8 — Table S5. Specific activities of the GH5 enzymes. (DOCX 14 kb) [file 40168_2018_421_MOESM8_ESM.docx]

**Table S5. Specific activities of the GH5 enzymes.** To assay endo*-*cellulase activity, enzymes were incubated with 1% (w/v) CMC or barley β-glucan (β-glc) at various enzyme concentrations in 20 mM citrate buffer, pH 5.5. Samples were taken at various time-points to produce progress curves, and glucose equivalents were measured by the DNS-assay (2). Cel5C_Cwt and Cel5C_CR displayed too low activity on CMC to produce progress curves under the conditions tested, and were instead characterized on β-glucan. Specific activities are given in U/µmol protein, from time-points and enzyme concentrations that were determined to generate approximate initial rate conditions. 1 U of activity was defined as the release of 1µmol of glucose equivalents per minute. Cel5A_N: N-terminal domain of Cel5A, Cel5A_wt: full-length Cel5A, Cel5A_C: C-terminal domain of Cel5A, Cel5B: full length Cel5B, Cel5C_wt: full-length Cel5C, Cel5C_R: full-length Cel5C with restored catalytic-site, Cel5C_Cwt: Cel5C C-terminal domain, Cel5C_CR, Cel5C C-terminal domain with restored catalytic-site, Cel5C_N: N-terminal domain of Cel5C, Cel5D: full-length Cel5D.

|  | **Enzyme concentration (nM)** | | **Time (min)** | **Sp. Activity (U/µmol)** |
| --- | --- | --- | --- | --- |
| Cel5A_N | | 1000 | 10 | 136 |
| Cel5A_wt | | 1000 | 10 | 123 |
| Cel5A_C | | 1000 | 60 | 0 |
| Cel5B | | 500 | 5 | 750 |
| Cel5C_wt | | 50 | 2.5 | 11 465 |
| Cel5C_R | | 50 | 15 | 1 144 |
| Cel5C_N | | 50 | 2.5 | 13 889 |
| Cel5D | | 25 | 5 | 10 087 |
|  | |  |  |  |
| Cel5C_Cwt (β-glc) | | 1000 | 15 | 68 |
| Cel5C_CR (β-glc) | | 1000 | 15 | 119 |
