# Supplementary material for: “Candidatus Paraporphyromonas polyenzymogenes” encodes multi-modular cellulases linked to the type IX secretion system
Source: Microbiome. 2018 Mar 1;6:44. doi: 10.1186/s40168-018-0421-8 (PMC5831590; doi:10.1186/s40168-018-0421-8)
Supplement: Supplementary file 9 — Figure S4. Multiple alignment of GH5 domains. (DOCX 39 kb) [file 40168_2018_421_MOESM9_ESM.docx]

| Cel5A_N | - | - | - | - | - | - | - | - | - | - | - | - | - | - | - | - | - | - | - | - | - | - | - | M | A | D | V | S | D | N | Y | I | E | K | W | G | R | L | K | L | V | G | I | Q | L | S | S | E | N | G | E | A | I | Q | L | K | G | W | S | S | F | -38 |
| --- | --- | --- | --- | --- | --- | --- | --- | --- | --- | --- | --- | --- | --- | --- | --- | --- | --- | --- | --- | --- | --- | --- | --- | --- | --- | --- | --- | --- | --- | --- | --- | --- | --- | --- | --- | --- | --- | --- | --- | --- | --- | --- | --- | --- | --- | --- | --- | --- | --- | --- | --- | --- | --- | --- | --- | --- | --- | --- | --- | --- | --- | --- |
| Cel5B | - | M | G | G | G | G | N | N | A | K | - | - | - | - | - | - | - | - | Q | K | K | S | V | S | P | Y | P | E | N | S | P | V | A | K | Y | G | R | L | Q | V | K | D | L | Q | L | C | D | K | D | G | N | P | V | Q | L | A | G | M | S | T | M | -52 |
| Cel5C_N | - | M | G | G | Y | D | L | N | E | E | E | M | E | Q | R | V | S | P | A | P | N | A | G | Y | T | Y | P | E | G | S | P | V | Y | H | N | G | K | L | S | V | Q | G | T | Q | M | V | S | E | C | G | K | P | V | Q | L | R | G | M | S | S | H | -60 |
| Cel5C_C | - | - | - | - | - | - | M | S | I | P | T | Q | E | Y | V | E | I | V | P | A | M | N | K | V | S | Y | P | K | G | S | P | I | Y | H | N | G | Q | L | H | V | Q | N | G | R | L | T | N | E | C | D | F | D | V | Q | L | R | G | V | S | S | D | -55 |
| Cel5C_CR | - | - | - | - | - | - | M | S | I | P | T | Q | E | Y | V | E | I | V | P | A | M | N | K | V | S | Y | P | K | G | S | P | I | Y | H | N | G | Q | L | H | V | Q | N | G | R | L | T | N | E | C | D | F | D | V | Q | L | R | G | V | S | S | D | -55 |
| Cel5D | - | M | G | G | Y | D | M | N | E | K | G | T | G | H | R | V | S | S | A | P | N | V | R | Y | N | Y | P | V | G | S | P | V | Y | Y | N | G | K | L | S | V | Q | G | T | Q | M | V | N | E | C | G | K | P | V | Q | L | K | G | M | S | S | H | -60 |
| I |  |  |  |  |  |  |  |  |  |  |  |  |  |  |  |  |  |  |  |  |  |  |  |  |  |  |  |  |  |  |  |  |  |  |  |  |  |  |  |  |  |  |  |  |  |  |  |  |  |  |  |  |  |  |  |  |  |  |  |  |  |  |
|  |  |  |  |  |  |  |  |  |  |  |  |  |  |  |  |  |  |  |  |  |  |  |  |  |  |  |  |  |  |  |  |  |  |  |  |  |  |  |  |  |  |  |  |  |  |  |  |  |  |  |  |  |  |  |  |  |  |  |  |  |  |  |
| Cel5A_N | - | G | F | Y | - | G | E | N | C | L | T | S | A | N | D | L | E | S | M | K | G | A | G | A | N | C | V | R | I | A | R | Y | L | G | N | G | S | G | S | I | D | D | N | G | I | K | N | W | - | - | - | - | - | - | - | - | - | - | - | - | - | -84 |
| Cel5B | - | G | W | Q | W | C | G | D | C | Y | T | K | E | S | I | K | T | M | V | E | E | W | G | I | N | V | L | R | L | A | M | Y | V | E | E | - | G | G | - | Y | N | T | N | - | - | - | - | - | - | - | P | I | G | F | K | Q | R | M | C | E | M | -103 |
| Cel5C_N | - | G | L | A | W | F | P | K | C | Y | T | E | A | S | L | T | A | L | V | K | D | W | N | I | D | I | F | R | L | A | I | Y | T | H | E | W | G | G | - | Y | T | T | - | - | - | N | Q | W | K | S | K | D | D | Y | N | A | Y | I | D | N | M | -116 |
| Cel5C_C | - | N | M | S | I | Y | T | R | C | Y | S | T | S | S | L | T | A | L | A | N | D | W | N | A | S | L | F | R | I | S | V | N | T | N | G | K | G | G | - | Y | C | V | N | G | S | D | Q | W | L | S | M | Y | D | Y | N | D | K | V | D | E | L | -114 |
| Cel5C_CR | - | N | M | S | I | Y | T | R | C | Y | S | T | S | S | L | T | A | L | A | N | D | W | N | A | S | L | F | R | I | S | V | N | T | N | G | K | G | G | - | Y | C | V | N | G | S | D | Q | W | L | S | M | Y | D | Y | N | D | K | V | D | E | L | -114 |
| Cel5D | - | G | L | A | W | F | P | Q | C | Y | T | E | E | S | L | S | V | L | V | N | D | W | H | I | D | I | F | R | L | A | I | Y | T | H | E | K | G | G | - | Y | C | K | T | D | G | T | Q | W | K | S | K | E | D | Y | N | A | Y | I | D | E | L | -119 |
| I |  |  |  |  |  |  |  |  |  |  |  |  |  |  |  |  |  |  |  |  |  |  |  |  |  |  |  | ▲ | A | r | g |  |  |  |  |  |  |  |  |  |  |  |  |  |  |  |  |  |  |  |  |  |  |  |  |  |  |  |  |  |  |  |
|  |  |  |  |  |  |  |  |  |  |  |  |  |  |  |  |  |  |  |  |  |  |  |  |  |  |  |  |  |  |  |  |  |  |  |  |  |  |  |  |  |  |  |  |  |  |  |  |  |  |  |  |  |  |  |  |  |  |  |  |  | * |  |
| Cel5A_N | - | M | S | W | T | A | Q | K | G | M | Y | C | V | I | D | W | H | I | L | E | A | A | N | G | D | G | N | P | G | K | Y | T | N | D | A | K | N | F | F | R | M | V | A | Q | E | V | A | D | K | K | Y | K | H | I | I | Y | E | L | C | N | E | -144 |
| Cel5B | - | I | D | I | C | G | E | L | G | I | Y | C | I | V | D | W | H | I | L | T | P | G | N | - | - | P | - | L | D | S | K | Y | G | G | A | K | E | F | F | S | F | I | S | K | K | Y | A | - | - | N | K | E | H | L | L | Y | E | I | C | N | E | -158 |
| Cel5C_N | - | V | D | I | C | A | K | L | G | I | Y | C | I | I | D | W | H | V | L | N | D | G | S | - | - | G | D | P | N | Y | T | L | D | D | A | I | P | F | W | D | Y | M | S | A | K | H | K | - | - | D | D | K | H | V | L | Y | E | I | C | N | E | -172 |
| Cel5C_C | - | V | R | L | C | G | M | R | G | L | Y | C | V | V | D | W | H | L | N | E | - | - | G | - | - | G | D | P | N | A | H | L | K | E | A | T | V | F | W | R | H | M | A | Q | R | Y | T | - | - | K | F | T | H | V | I | F | E | I | C | D | N | -168 |
| Cel5C_CR | - | V | R | L | C | G | M | R | G | L | Y | C | V | V | D | W | H | L | N | E | - | - | G | - | - | G | D | P | N | A | H | L | K | E | A | T | V | F | W | R | H | M | A | Q | R | Y | T | - | - | K | F | T | H | V | I | F | E | I | C | N | E | -168 |
| Cel5D | - | V | A | I | C | G | K | L | G | I | Y | C | I | I | D | W | H | I | L | Q | E | G | S | - | - | G | N | P | K | N | T | L | D | D | A | I | P | F | W | E | Y | M | S | A | K | H | K | - | - | D | D | K | H | V | L | Y | E | I | C | N | E | -175 |
| I |  |  |  |  |  |  |  |  |  |  |  |  |  |  |  |  | ▲ | Hi | s |  |  |  |  |  |  |  |  |  |  |  |  |  |  |  |  |  |  |  |  |  |  |  |  |  |  |  |  |  |  |  |  |  |  |  |  |  | A | s | n | ▲ | ▲ | Glu |
|  |  |  |  |  |  |  |  |  |  |  |  |  |  |  |  |  |  |  |  |  |  |  |  |  |  |  |  |  |  |  |  |  |  |  |  |  |  |  |  |  |  |  |  |  |  |  |  |  |  |  |  |  |  |  |  |  |  |  |  |  |  |  |
| Cel5A_N | - | P | S | G | - | - | - | - | - | - | - | - | - | - | - | - | - | - | - | - | - | - | V | G | W | G | T | I | K | S | Y | A | E | D | V | I | K | T | I | V | A | I | D | - | - | - | - | K | N | K | P | V | V | I | V | G | T | P | N | W | D | -182 |
| Cel5B | - | P | N | N | C | L | E | K | G | D | P | I | H | P | W | V | C | T | K | E | T | N | V | T | W | D | M | I | A | D | Y | A | D | E | I | I | P | A | I | Q | G | N | Y | D | S | L | K | V | S | H | P | I | V | I | V | G | T | P | Q | W | D | -218 |
| Cel5C_N | - | P | N | G | F | - | - | - | - | - | - | - | - | - | - | - | - | - | - | - | - | D | V | K | W | A | D | V | K | E | Y | A | E | A | V | I | P | V | I | R | K | N | D | - | - | - | - | - | P | D | K | I | I | I | C | G | T | P | T | W | S | -211 |
| Cel5C_C | - | A | S | G | - | - | - | - | - | - | - | - | - | - | - | - | - | - | - | - | - | - | V | E | W | S | A | I | K | S | Y | A | D | S | M | I | A | L | I | R | Q | F | D | - | - | - | - | - | K | N | K | V | I | I | C | G | T | P | S | N | D | -205 |
| Cel5C_CR | - | P | S | G | - | - | - | - | - | - | - | - | - | - | - | - | - | - | - | - | - | - | V | E | W | S | A | I | K | S | Y | A | D | S | M | I | A | L | I | R | Q | F | D | - | - | - | - | - | K | N | K | V | I | I | C | G | T | P | S | N | D | -205 |
| Cel5D | - | P | N | G | F | - | - | - | - | - | - | - | - | - | - | - | - | - | - | - | - | M | V | R | W | S | D | V | K | E | Y | A | D | K | V | I | P | V | I | R | A | N | D | - | - | - | - | - | P | D | K | I | I | I | C | G | T | P | M | W | S | -214 |
| I |  |  |  |  |  |  |  |  |  |  |  |  |  |  |  |  |  |  |  |  |  |  |  |  |  |  |  |  |  |  |  |  |  |  |  |  |  |  |  |  |  |  |  |  |  |  |  |  |  |  |  |  |  |  |  |  |  |  |  |  |  |  |
|  |  |  |  |  |  |  |  |  |  |  |  |  |  |  |  |  |  |  |  |  |  |  |  |  |  |  |  |  |  |  |  |  |  |  |  |  |  |  |  |  |  |  |  |  |  |  |  |  |  |  |  |  |  |  |  |  |  |  |  |  |  |  |
| Cel5A_N | - | Q | Y | I | Y | S | Q | V | A | S | K | G | - | - | - | - | - | - | - | - | - | - | - | - | - | - | D | L | I | N | T | N | D | A | Y | V | M | Y | A | F | H | L | Y | A | N | E | A | A | H | V | G | L | E | S | S | E | - | - | - | - | - | -223 |
| Cel5B | - | Q | L | V | D | A | C | L | K | E | G | M | Y | Q | G | N | G | K | D | L | C | D | S | L | P | A | R | D | A | R | L | K | H | D | N | V | M | Y | A | F | H | F | Y | A | K | E | H | N | E | G | - | F | E | K | D | G | K | P | D | Y | Y | -277 |
| Cel5C_N | - | Q | D | V | D | L | A | A | Q | - | - | - | - | - | - | - | - | - | - | - | - | - | - | - | - | - | - | - | D | P | L | S | Y | D | N | V | M | Y | T | L | H | F | Y | S | G | T | H | T | Q | Y | - | L | R | D | K | A | Q | V | - | - | - | -248 |
| Cel5C_C | - | R | E | W | S | S | V | I | S | - | - | - | - | - | - | - | - | - | - | - | - | - | - | - | - | - | - | - | N | P | L | S | D | S | N | V | M | Y | A | L | H | F | T | V | G | T | D | G | Q | S | - | L | R | D | K | A | D | A | - | - | - | -242 |
| Cel5C_CR | - | R | E | W | S | S | V | I | S | - | - | - | - | - | - | - | - | - | - | - | - | - | - | - | - | - | - | - | N | P | L | S | D | S | N | V | M | Y | A | L | H | F | T | V | G | T | D | G | Q | S | - | L | R | D | K | A | D | A | - | - | - | -242 |
| Cel5D | - | Q | D | V | D | L | A | S | Q | - | - | - | - | - | - | - | - | - | - | - | - | - | - | - | - | - | - | - | N | P | L | S | Y | N | N | V | M | Y | T | L | H | F | Y | S | G | D | H | F | Q | S | - | L | R | D | K | A | Q | T | - | - | - | -251 |
| I |  |  |  |  |  |  |  |  |  |  |  |  |  |  |  |  |  |  |  |  |  |  |  |  |  |  |  |  |  |  |  |  |  |  |  |  |  |  | H | is | ▲ |  | ▲ | T | y | r |  |  |  |  |  |  |  |  |  |  |  |  |  |  |  |  |
|  |  |  |  |  |  |  |  |  |  |  |  |  |  |  |  |  |  |  |  | * |  |  |  |  |  |  |  |  |  |  |  |  |  |  |  |  |  |  |  |  |  |  |  |  |  |  |  |  |  |  |  |  |  |  |  |  |  |  |  |  |  |  |
| Cel5A_N | - | - | - | - | - | - | F | L | P | A | S | T | R | I | P | I | F | V | S | E | W | G | L | S | S | A | Q | P | E | K | R | G | S | Y | D | D | V | N | T | S | F | A | T | T | F | L | R | H | C | A | G | Y | D | G | C | G | Q | I | V | S | W | -278 |
| Cel5B | - | N | M | Y | A | Y | M | Y | D | V | L | G | K | L | P | V | F | C | S | E | F | G | L | C | E | A | N | G | N | G | E | - | - | - | - | - | L | D | P | D | R | T | D | K | W | L | L | L | L | S | G | N | N | A | G | K | Q | V | V | S | F | -332 |
| Cel5C_N | - | - | - | - | - | - | - | - | A | I | N | K | G | L | A | L | F | V | T | E | F | G | T | T | Q | A | S | G | D | G | G | - | - | - | - | - | V | Y | F | D | E | C | N | T | W | M | D | W | M | D | - | - | - | - | - | A | R | K | I | S | W | -291 |
| Cel5C_C | - | - | - | - | - | - | - | - | A | I | S | R | G | L | P | L | F | V | S | E | F | S | L | S | P | S | - | N | G | G | S | - | - | - | - | - | V | N | T | T | E | A | E | Q | W | I | T | W | M | K | - | - | - | - | - | N | Q | G | L | S | W | -284 |
| Cel5C_CR | - | - | - | - | - | - | - | - | A | I | S | R | G | L | P | L | F | V | S | E | F | S | L | S | P | S | - | N | G | G | S | - | - | - | - | - | V | N | T | T | E | A | E | Q | W | I | T | W | M | K | - | - | - | - | - | N | Q | G | L | S | W | -284 |
| Cel5D | - | - | - | - | - | - | - | - | A | L | N | N | G | A | A | I | F | V | T | E | F | G | T | T | K | A | S | G | D | G | G | - | - | - | - | - | V | F | L | D | E | C | N | R | W | M | E | W | M | N | - | - | - | - | - | E | R | K | I | S | W | -294 |
| I |  |  |  |  |  |  |  |  |  |  |  |  |  |  |  |  |  |  |  | ▲ | G | lu |  |  |  |  |  |  |  |  |  |  |  |  |  |  |  |  |  |  |  |  |  |  |  |  |  |  |  |  |  |  |  |  |  |  |  |  |  |  | ▲ | Trp |
|  |  |  |  |  |  |  |  |  |  |  |  |  |  |  |  |  |  |  |  |  |  |  |  |  |  |  |  |  |  |  |  |  |  |  |  |  |  |  |  |  |  |  |  |  |  |  |  |  |  |  |  |  |  |  |  |  |  |  |  |  |  |  |
| Cel5A_N | - | M | N | W | S | Y | G | M | K | R | E | G | S | S | T | F | K | D | R | C | - | G | - | - | - | - | G | E | L | S | P | S | G | K | F | I | Q | E | M | L | G | G | E | L | K | P | V | V | T | A | C | Y | G | G | I | C | F | - | - | - | - | -329 |
| Cel5B | - | C | N | W | S | F | S | D | N | E | R | S | S | S | A | L | N | P | G | A | C | A | R | E | A | W | N | D | V | T | P | S | G | D | Y | I | K | R | I | L | S | V | V | N | K | - | - | - | - | - | - | - | G | G | V | D | S | T | V | L | K | -385 |
| Cel5C_N | - | V | N | W | S | F | A | D | K | P | E | S | S | A | A | L | K | P | G | A | S | N | S | G | D | W | N | M | V | S | E | S | G | Q | Y | I | K | R | K | L | S | Q | P | K | S | Y | E | S | C | G | - | - | - | - | - | - | - | - | - | - | - | -340 |
| Cel5C_C | - | A | N | A | H | Y | A | D | G | N | D | L | N | S | M | L | L | S | G | A | C | G | T | K | D | W | N | S | V | S | V | A | G | E | Y | I | K | S | K | L | S | E | P | H | N | F | H | G | - | - | - | - | - | - | - | - | - | - | - | - | - | -331 |
| Cel5C_CR | - | A | N | A | H | Y | A | D | G | N | D | L | N | S | M | L | L | S | G | A | C | G | T | K | D | W | N | S | V | S | V | A | G | E | Y | I | K | S | K | L | S | E | P | H | N | F | H | G | - | - | - | - | - | - | - | - | - | - | - | - | - | -331 |
| Cel5D | - | V | N | W | S | F | S | D | K | A | E | S | S | A | A | L | Q | P | G | A | S | R | S | K | N | W | N | M | V | S | E | S | G | Q | Y | I | K | Q | M | L | S | Q | P | K | N | F | E | P | C | E | D | I | N | G | V | E | D | N | L | L | D | -354 |
| I |  |  |  |  |  |  |  |  |  |  |  |  |  |  |  |  |  |  |  |  |  |  |  |  |  |  |  |  |  |  |  |  |  |  |  |  |  |  |  |  |  |  |  |  |  |  |  |  |  |  |  |  |  |  |  |  |  |  |  |  |  |  |

**Figure S4. Multiple alignment of GH5 domains.** The catalytic domain sequences of the *Ca.* P. polyenzymogenes cellulases were aligned using Clustal Omega and visualized using Multiple Align Show [1, 2]. The catalytic site mutation in Cel5C_C is highlighted in yellow, and the restored sequence is included in the alignment directly below. The catalytic glutamates are marked by *, and amino acids corresponding to residues that are highly conserved in GH5s [3] are indicated by ▲. ≥80% conservation of residues is indicated by shading in dark green, whereas positions with less conservation of identity but conservation of similarity are shaded in light green.

1. Stothard P: **The sequence manipulation suite: JavaScript programs for analyzing and formatting protein and DNA sequences.** *Biotechniques* 2000, **28**:1102-1104.

2. Sievers F, Wilm A, Dineen D, Gibson TJ, Karplus K, Li WL, R., McWilliam H, Remmert MS, J., Thompson JD, Higgins DG: **Fast, scalable generation of high-quality protein multiple sequence alignments using Clustal Omega.** *Mol Syst Biol* 2011, **7**:539.

3. Bianchetti CM, Brumm P, Smith RW, Dyer K, Hura GL, Rutkoski TJ, Phillips GNJ: **Structure, dynamics, and specificity of endoglucanase D from *Clostridium cellulovorans*.** *J Mol Biol* 2013, **425**:4267-4285.
