# Supplementary material for: “Candidatus Paraporphyromonas polyenzymogenes” encodes multi-modular cellulases linked to the type IX secretion system
Source: Microbiome. 2018 Mar 1;6:44. doi: 10.1186/s40168-018-0421-8 (PMC5831590; doi:10.1186/s40168-018-0421-8)
Supplement: Supplementary file 10 — Supplemental text Does the C-terminal Cel5C domain contain a mutation in its active site?. (DOCX 20 kb) [file 40168_2018_421_MOESM10_ESM.docx]

**SUPPLEMENTAL TEXT S1**

**Does the C-terminal Cel5C domain contain a mutation in its active site?**

Based on a multiple sequence alignment of the individual catalytic domains, the C-terminal GH5 domain of Cel5C (Cel5C_C) was suspected to contain a mutation in its active site, since the catalytic glutamate acid/base and its two adjacent amino acids were mutated from the conserved Asn-Glu-Pro to Asp-Asn-Ala (**Additional file 9:** **Figure S4**). To assess whether this was a sequencing/assembly error, we expressed both the “wild-type” genes containing the mutation (Cel5C_wt and Cel5C_C), as well as corrected versions where the Asn-Glu-Pro sequence was restored for both the full-length and individual domains (Cel5C_R and Cel5C_CR, respectively). Restoration of the active site increased activity of the individual domain on β-glucan, indicating that the reported sequence was erroneous, although the increase was modest (from 68 U/μmol for Cel5C_C to 119 U/μmol for Cel5C_CR).

Restoring the catalytic site in Cel5C_C was expected to increase, or at least not change, the CMCase activity of full-length Cel5C. Surprisingly, the activity of Cel5C_R (1144 U/μmol) was about ten times lower compared to Cel5C_wt (11465 U/μmol) or Cel5C_N (13889 U/μmol) (**Additional file 8: Table S5**). Also on Avicel, Cel5C_R produced substantially lower amounts of products than Cel5C_N (**Additional file 11:** **Figure S5**). The negative impact of the restored C-terminal domain on the activity of N-terminal domain that was observed in the full length protein was much less noticeable when adding equimolar amounts Cel5C_N and Cel5C_CR, with the latter combination giving almost the same activity as Cel5C_N (**Additional file 11: Figure S5**).

Both Cel5C_R and Cel5C_CR were difficult to purify, producing almost exclusively inclusion bodies in *E. coli.* The small amount of soluble protein obtained could therefore be misfolded, which could impact activity [1, 2]. Cel5C was also found to contain two motifs of the phylum-wide *Bacteroidetes* O-glycosylation site, one in the linker region between the GH5 domains, and one in Cel5C_C [3], suggesting that glycosylation could be necessary for proper folding of the wild-type protein. Similar circumstances have been observed with the highly active multi-modular cellulase *Cb*CelA from *Caldicellulosiruptor bescii,* which has low activity when expressed heterologously, but outperform commercial cellulase mixtures when purified from native culture supernatant [4, 5]. Chung and colleagues recently showed that the enzyme is glycosylated when expressed and secreted homologously, restoring function compared to non-glycosylated protein [6]. It remains to be seen whether the surprising results obtained in this work are due to folding and/or glycosylation issues, whether the two GH5 domains in Cel5C interact in a way that we do not yet understand, and whether the “mutation” in Cel5C_C is biologically relevant. In principle, it is conceivable that restoration of the “mutation” in Cel5C_C leads to a change in how full length Cel5C binds to the substrate, which could leave Cel5C_N incapable of interacting productively with the substrate.

**Hemicellulase activity of Cel5A-D**

In addition to cellulose, Cel5A-D all displayed activity on hemicellulose substrates that contain β-1,4-glycosidic glucan backbones (**Additional file 15: Figure S9**). Activity was seen on the mixed linkage β-1,3/β-1,4 glucan substrates barley β-glucan and lichenin, as well as konjac glucomannan (a linear polymer of β(1,4) linked mannose and glucose with approximately 60% mannose), but not on tamarind xyloglucan, wheat arabinoxylan, xylan, ivory nut mannan, or galactomannans. The enzymes also did not display activity on Pachyman, which consists of glucose units linked by β-1,3 glycosidic bonds, confirming the specificity for β-1,4 bonds. Cel5C_Cwt was the only GH5 domain not active on Konjac glucomannan, suggesting a specificity towards substrates containing only glucose units in the backbone. Compared to wild-type Cel5C_C, Cel5C_CR demonstrated increased catalytic efficiency on Lichenan (3.5-fold), which has a higher β-1,3:β-1;4 ratio than Barley β-glucan.

1. González-Montalbán N, García-Fruitós E, Villaverde A: **Recombinant protein solubility - does more mean better?** *Nat Biotechnol* 2007, **25**:718-720.

2. Martínez-Alonso M, González-Montalbán N, García-Fruitós E, Villaverde A: **The Functional quality of soluble recombinant polypeptides produced in <i>Escherichia coli </i>is defined by a wide conformational spectrum.** *Appl Environ Microb* 2008, **74**:7431-7433.

3. Coyne MJ, Fletcher CM, Chatzidaki-Livanis M, Posch G, Schaffer C, Comstock LE: **Phylum-wide general protein O-glycosylation system of the Bacteroidetes.** *Mol Microbiol* 2013, **88**:772-783.

4. Brunecky R, Alahuhta M, Xu Q, Donohoe BS, Crowley MF, Kataeva IA, Yang SJ, Resch MG, Adams MW, Lunin VV *et al*: **Revealing nature's cellulase diversity: the digestion mechanism of *Caldicellulosiruptor bescii* CelA.** *Science* 2013, **342**:1513-1516.

5. Yi Z, Su X, Revindran V, Mackie RI, Cann I: **Molecular and Biochemical Analyses of CbCel9A/Cel48A, a Highly Secreted Multi-Modular Cellulase by *Caldicellulosiruptor bescii* during Growth on Crystalline Cellulose.** *PLoS One* 2013, **8**:e84172.

6. Chung D, Young J, Bomble YJ, Wall TAV, Groom J, Himmel ME, Westpheling J: **Homologous expression of the *Caldicellulosiruptor bescii* CelA reveals that the extracellular protein is glycosylated.** *PLoS One* 2015, **10**:1-11.
