# Supplementary material for: “Candidatus Paraporphyromonas polyenzymogenes” encodes multi-modular cellulases linked to the type IX secretion system
Source: Microbiome. 2018 Mar 1;6:44. doi: 10.1186/s40168-018-0421-8 (PMC5831590; doi:10.1186/s40168-018-0421-8)
Supplement: Supplementary file 11 — Figure S5. Comparison of individual Cel5C domains and the two domains combined. (DOCX 40 kb) [file 40168_2018_421_MOESM11_ESM.docx]

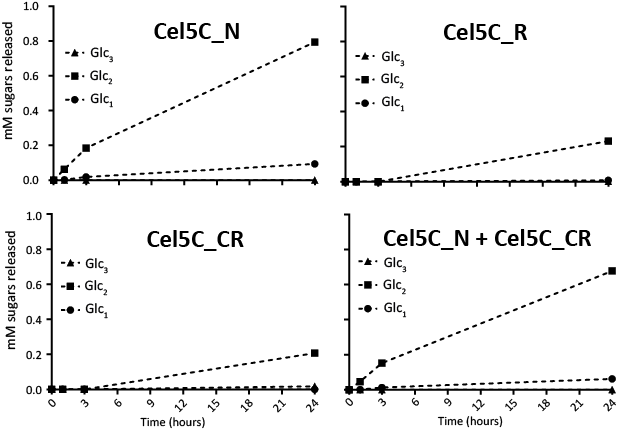


**Figure S5.** Comparison of individual Cel5C domains and the two domains combined. Initial experiments with wild-type Cel5C_C showed no activity on Avicel, and since we showed that the catalytic restoration of Cel5C_C increased the activity of the domain, only the restored versions of the Cel5C proteins were included in this assay.
