# Supplementary material for: “Candidatus Paraporphyromonas polyenzymogenes” encodes multi-modular cellulases linked to the type IX secretion system
Source: Microbiome. 2018 Mar 1;6:44. doi: 10.1186/s40168-018-0421-8 (PMC5831590; doi:10.1186/s40168-018-0421-8)
Supplement: Supplementary file 12 — Figure S6. Cel5A_C binding to Avicel cellulose. (DOCX 36 kb) [file 40168_2018_421_MOESM12_ESM.docx]

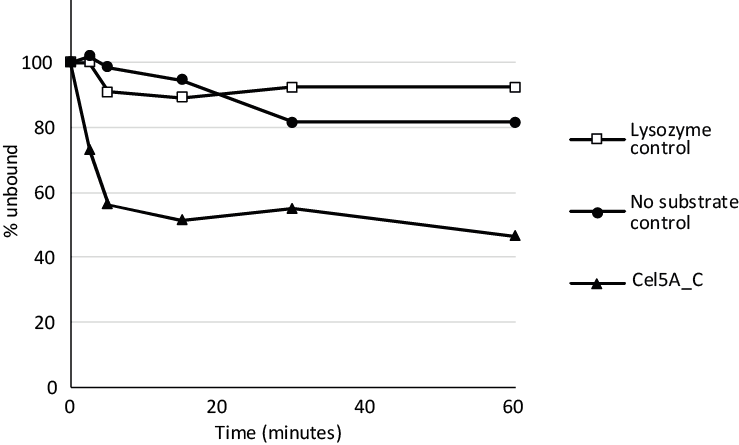


**Figure S6: Cel5A_C binding to Avicel cellulose.** To assess whether the Cel5A_C protein could bind to cellulose 0.08 mg/ml protein was incubated with 1% (w/v) Avicel at 40 ºC with 1000 rpm vertical shaking. Values are given as % unbound protein.
