# Supplementary material for: “Candidatus Paraporphyromonas polyenzymogenes” encodes multi-modular cellulases linked to the type IX secretion system
Source: Microbiome. 2018 Mar 1;6:44. doi: 10.1186/s40168-018-0421-8 (PMC5831590; doi:10.1186/s40168-018-0421-8)
Supplement: Supplementary file 13 — Figure S7. Degradation of Glc(5) and Glc(6) by GH5 enzymes. (DOCX 540 kb) [file 40168_2018_421_MOESM13_ESM.docx]

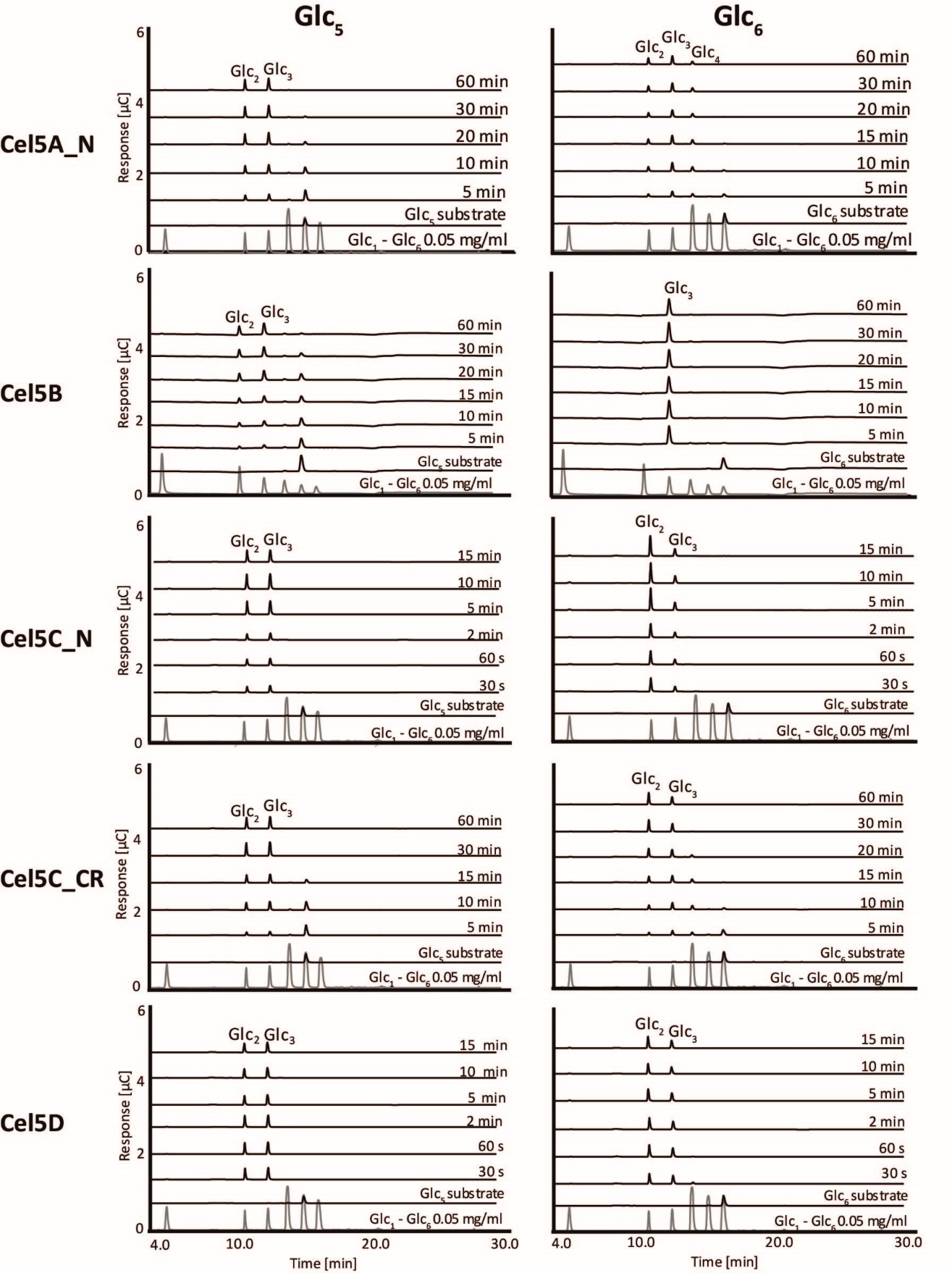


**Figure S7. Degradation of Glc_(5)_ and Glc_(6)_ by GH5 enzymes.** 0.1 mg/ml cellodextrins were incubated with 0.25µM enzyme in 20mM citrate buffer pH 5.5. Samples were taken at indicated intervals and the reaction was stopped by adding NaOH to 0.1M. Products were analyzed using HPAEC-PAD with cellodextrins as standards.
