# Supplementary material for: “Candidatus Paraporphyromonas polyenzymogenes” encodes multi-modular cellulases linked to the type IX secretion system
Source: Microbiome. 2018 Mar 1;6:44. doi: 10.1186/s40168-018-0421-8 (PMC5831590; doi:10.1186/s40168-018-0421-8)
Supplement: Supplementary file 14 — Figure S8. Degradation of cellodextrins by the GH3 β-glucosidase. (DOCX 259 kb) [file 40168_2018_421_MOESM14_ESM.docx]

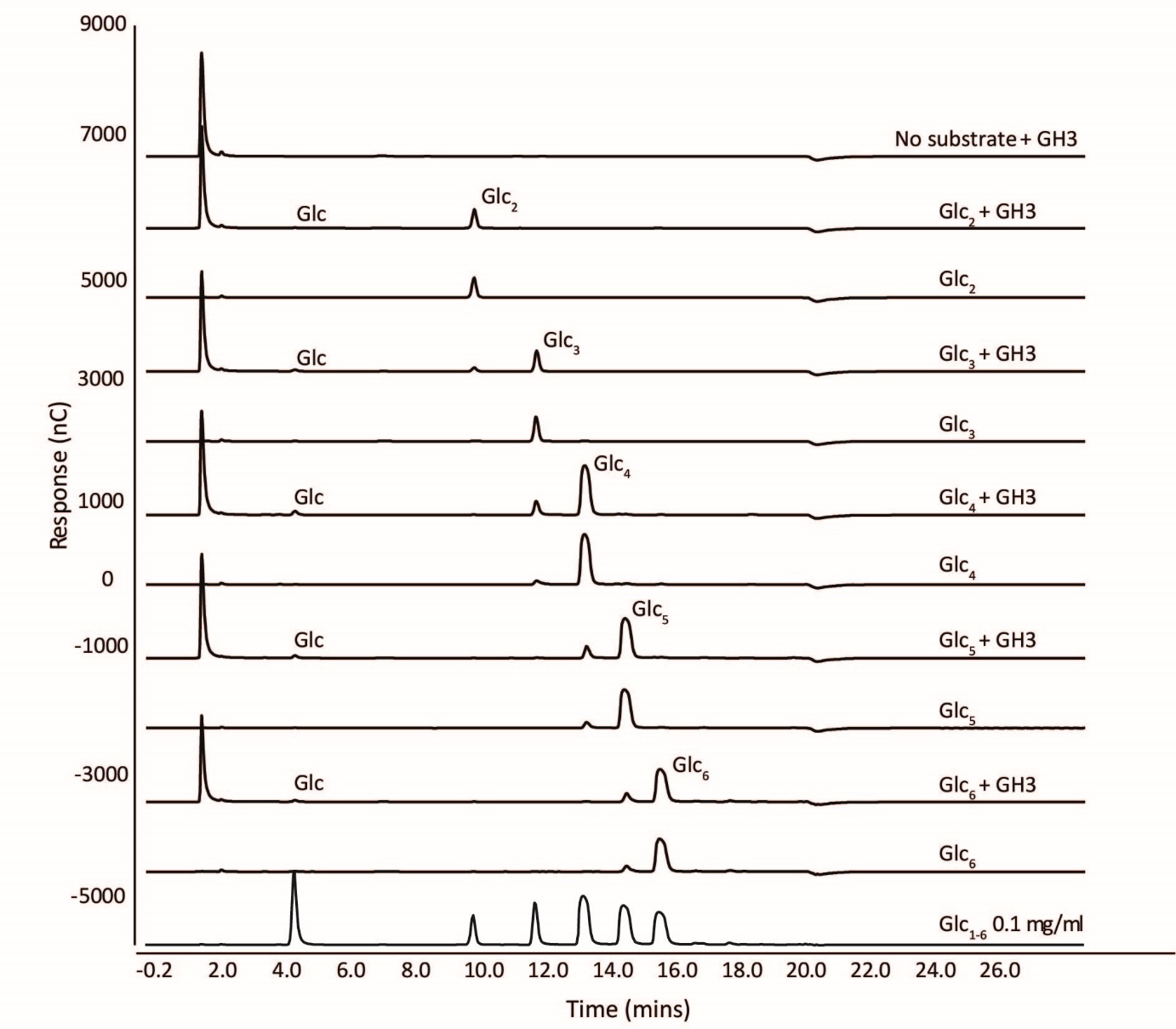


**Figure S8. Degradation of cellodextrins by the GH3 β-glucosidase.** To assay the cellodextrin activity of the GH3 in the cellulose cluster, the enzyme (1 µM) was incubated with cellodextrins of various lengths (0.1 mg/ml) in Citrate buffer (20 mM, pH 5.5) at 40 °C. Products were analyzed after 25 hours by HPAEC-PAD after stopping the reaction by addition of NaOH to 0.1M. The GH3 enzyme released glucose from all assayed cellodextrins.
