# Supplementary material for: “Candidatus Paraporphyromonas polyenzymogenes” encodes multi-modular cellulases linked to the type IX secretion system
Source: Microbiome. 2018 Mar 1;6:44. doi: 10.1186/s40168-018-0421-8 (PMC5831590; doi:10.1186/s40168-018-0421-8)
Supplement: Supplementary file 15 — Figure S9. Hemicellulose activities of GH5 cellulases. (DOCX 474 kb) [file 40168_2018_421_MOESM15_ESM.docx]

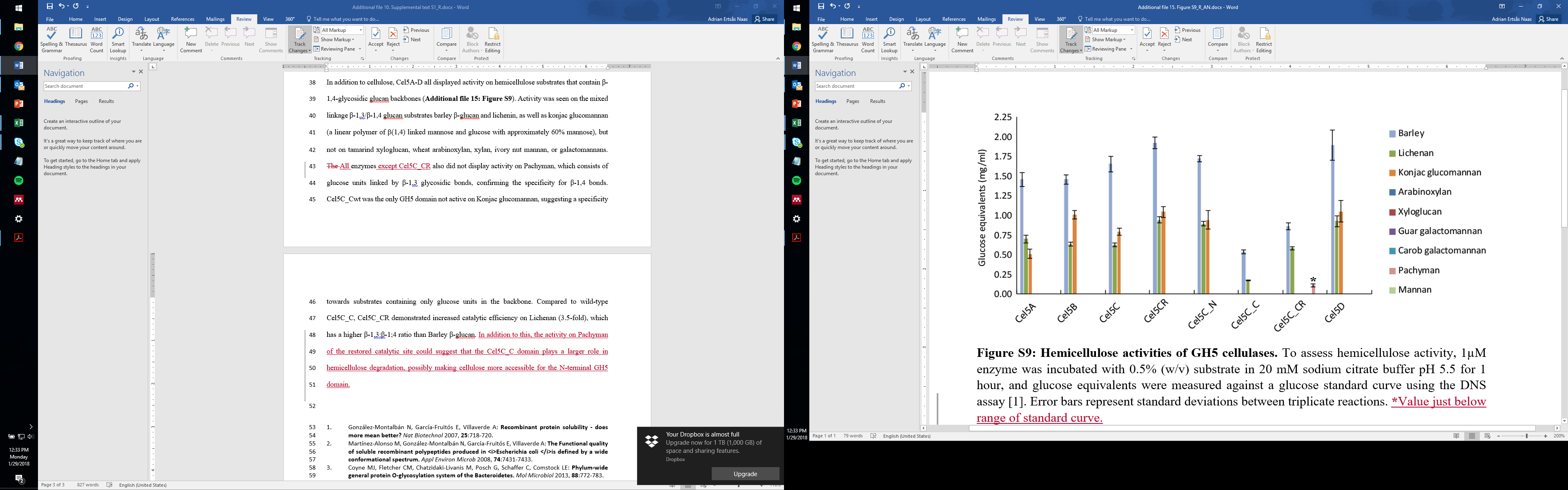


**Figure S9: Hemicellulose activities of GH5 cellulases.** To assess hemicellulose activity, 1µM enzyme was incubated with 0.5% (w/v) substrate in 20 mM sodium citrate buffer pH 5.5 for 1 hour, and glucose equivalents were measured against a glucose standard curve using the DNS assay [1]. Error bars represent standard deviations between triplicate reactions. *****Value just below range of standard curve.

1. Miller GL: **Use of dinitrosalicylic acid reagent for determination of reducing sugar.** *Anal Chem* 1959, **31**:426–428.
