# Supplementary material for: “Candidatus Paraporphyromonas polyenzymogenes” encodes multi-modular cellulases linked to the type IX secretion system
Source: Microbiome. 2018 Mar 1;6:44. doi: 10.1186/s40168-018-0421-8 (PMC5831590; doi:10.1186/s40168-018-0421-8)
Supplement: Supplementary file 16 — Figure S10. Crystal structure of Cel5C_N. (DOCX 1145 kb) [file 40168_2018_421_MOESM16_ESM.docx]

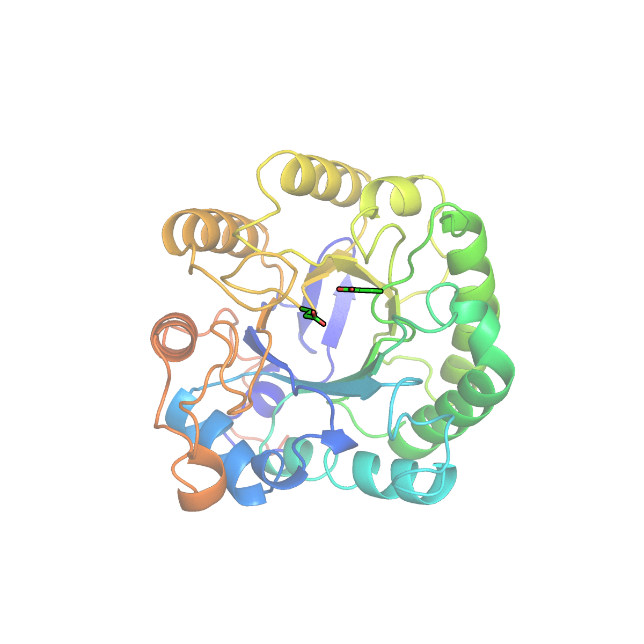


**E196**

**E284**


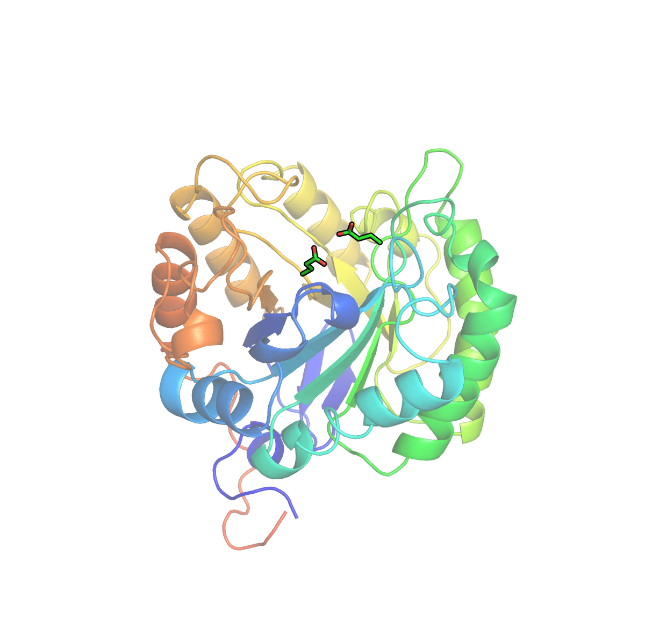


**E196**

**E284**

-45º

**Figure S10. Crystal structure of Cel5C_N. A**: Cartoon representation of the structure, seen from above the active-site cleft, and -45º rotated. The conserved catalytic glutamic acid side-chains are shown as stick models. Figures were created using The PyMOL Molecular Graphics System, Version 1.3 Schrödinger, LLC.
