# Supplementary material for: “Candidatus Paraporphyromonas polyenzymogenes” encodes multi-modular cellulases linked to the type IX secretion system
Source: Microbiome. 2018 Mar 1;6:44. doi: 10.1186/s40168-018-0421-8 (PMC5831590; doi:10.1186/s40168-018-0421-8)
Supplement: Supplementary file 17 — Table S6. Data collection and refinement statistics for the crystal structure of Cel5C_N. (DOCX 14 kb) [file 40168_2018_421_MOESM17_ESM.docx]

**Table S6:** Data Collection and Refinement Statistics for the crystal structure of Cel5C_N.

| **PDB ID** | 5WH8 |
| --- | --- |
| **Resolution range (Å)^1^** | 27.57 - 1.57 (1.626 - 1.57) |
| **Space group** | P 2_1_ 2_1_ 2_1_ |
| **Unit cell** | 49.21 75.5 88.83 90 90 90 |
| **Total reflections^1^** | 273948 (26343) |
| **Unique reflections^1^** | 46380 (4564) |
| **Multiplicity^1^** | 5.9 (5.8) |
| **Completeness (%)^1^** | 98.76 (99.00) |
| **Mean I/sigma(I)^1^** | 19.10 (3.02) |
| **Wilson B-factor** | 13.82 |
| **R-merge^1^** | 0.06999 (0.8158) |
| **R-meas** | 0.07694 |
| **CC1/2^1^** | 0.999 (0.854) |
| **CC*^1^** | 1 (0.96) |
| ***R*-work^1^** | 0.1448 (0.2854) |
| ***R*-free^1^** | 0.1740 (0.3272) |
| **Number of non-hydrogen atoms** | 2920 |
| **macromolecules** | 2605 |
| **ligands** | 96 |
| **water** | 219 |
| **Protein residues** | 323 |
| **RMS (bonds, Å)** | 0.01 |
| **RMS (angles, degrees)** | 1.35 |
| **Ramachandran favored (%)** | 97 |
| **Ramachandran allowed (%)** | 0 |
| **Ramachandran outliers (%)** | 0.31 |
| **Clashscore** | 0.95 |
| **Average B-factor** | 16.3 |
| **macromolecules** | 14.9 |
| **ligands** | 32.2 |
| **solvent** | 26.8 |

^1^ Values in parenthesis are for the highest resolution shell.
