# Supplementary material for: “Candidatus Paraporphyromonas polyenzymogenes” encodes multi-modular cellulases linked to the type IX secretion system
Source: Microbiome. 2018 Mar 1;6:44. doi: 10.1186/s40168-018-0421-8 (PMC5831590; doi:10.1186/s40168-018-0421-8)
Supplement: Supplementary file 18 — Table S7. Expression constructs used in this study. (DOCX 13 kb) [file 40168_2018_421_MOESM18_ESM.docx]

**Table S7. Expression constructs used in this study.** Proteins were expressed and purified as described in Materials and Methods. Accession numbers refer to gene-ids in IMG/MER Genome id 2061766007. His_6_; Histidine-tag for IMAC purification, TEV; TEV-protease recognition site, MBP; Maltose-binding protein solubility tag.

| **Protein Name** | **Accession number** | **Amino acids** | **Expression vector** | **Purification tag** |
| --- | --- | --- | --- | --- |
| Cel5A_wt | _HiSeq_23331250 | 20-641 | pETite N-His^1^ | N-His_6_-TEV- |
| Cel5A_C | _HiSeq_23331250 | 20-347 | pETite N-His^1^ | N-His_6_-TEV- |
| Cel5A_N | _HiSeq_23331250 | 375-524 | pETite N-His^1^ | N-His_6_-MBP-TEV- |
| GH3 | _HiSeq_23331270 | 1-587 | pNIC-CH^2^ | -His_6_-C |
| Cel5B | _HiSeq_23331280 | 19-409 | pNIC-CH^2^ | -His_6_-C |
| Cel5C_wt | _HiSeq_23331320 | 26-781 | pNIC-CH^2^ | -His_6_-C |
| Cel5C_R | _HiSeq_23331320 | 26-712  (aa 532-534; DNA→NEP)* | pMA-T^3^ | N-His_6_-V5-TEV- |
| Cel5C_N | _HiSeq_23331320 | 26-364 | pNIC-CH^2^ | -His_6_-C |
| Cel5C_C | _HiSeq_23331320 | 367-696 | pNIC-CH^2^ | -His_6_-C |
| Cel5C_CR | _HiSeq_23331320 | 367-696  (aa 532-534; DNA→NEP)* | pNIC-CH^2^ | -His_6_-C |
| Cel5D | _HiSeq_23331330 | 26-383 | pNIC-CH^2^ | -His_6_-C |

* Three amino acids mutated to correspond to consensus sequence of the catalytic site, see text for details.

^1^ Expresso™ T7 Cloning and Expression System (Lucigen)

^2^ Addgene plasmid #26117

^3^ GeneArt Expression vector (Thermo Scientific)
